# Influence of Countercations on the NH_3_‐SCR of NO Using Supported Vanadium‐Containing Polyoxometalate Catalysts

**DOI:** 10.1002/chem.202502561

**Published:** 2025-11-02

**Authors:** Samrin Shaikh, Leonhard Schill, Thomas Krøier Rønne‐Nielsen, Mariusz Grzegorz Kubus, James N. McPherson, Andreas Pawlig, Huirong Li, Maximillian J. Poller, Susanne Mossin, Anders Riisager, Jakob Albert

**Affiliations:** ^1^ Institute of Technical and Macromolecular Chemistry Universität Hamburg Bundesstraße 45 20146 Hamburg Germany; ^2^ Department of Chemistry Technical University of Denmark Kemitorvet, Building 207, Kgs Lyngby 2800 Denmark

**Keywords:** Brønsted acidity, cation influence, K poisoning, NH_3_‐SCR, polyoxometalates (POM)

## Abstract

Anatase‐supported vanadium‐substituted Keggin‐type polyoxometalates (POMs) are promising alternative catalysts for the selective catalytic reduction (SCR) of NO_x_ with NH_3_ at low temperatures. Generally, alkali poisoning is a major deactivation mechanism in vanadium‐catalyzed SCR, but changing the counter cations in the POM catalyst might be a propitious strategy for enhancing the catalyst stability. In this study, the effect and the role of cation variation were investigated in detail by exchanging the protons from the most promising Keggin‐type HPA‐3 (H_6_PV_3_Mo_9_O_40_) catalyst dispersed on anatase support with cations such as Na^+^, K^+^, Cs^+^. The synthesized catalysts were characterized in‐depth using ICP‐OES/AAS, FTIR, TGA, NH_3_‐TPD, EPR, H_2_‐TPR, ED (electron diffraction) and XPS. The supported HPA‐3/TiO_2_ catalyst with a 7.5 wt. % loading was found to be the most active catalyst for the SCR reaction in the temperature range of 200–300 °C, providing nearly complete NO conversion at 300 °C with a relatively high first‐order rate constant. In comparison, the analogous alkali‐exchanged catalysts possessed lower activities, which by characterization was corroborated to a combined effect of lower acidity and altered redox property. The study reveals new insight into NH_3_‐SCR catalysts comprising redox‐active POMs, which so far mainly have been applied for homogeneously catalyzed reactions.

## Graphical abstract

At low temperatures, anatase‐supported H_6_PV_3_Mo_9_O_40_ (HPA‐3) POM catalysts were investigated for NH_3_‐SCR. Lower acidity and redox shifts caused decreased activity when Na⁺, K⁺, and Cs⁺ cations were substituted. According to the EPR studies, the supported catalyst's acidity and vanadium redox property work in concert to control the catalytic activity of POM‐supported on TiO_2_.

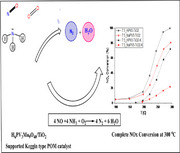

## 1 Introduction

Emission of NO_x_ gases (mostly NO and NO_2_) formed from combustion of hydrocarbons has since the mid‐20^th^ century caused major environmental issues such as, for example, photochemical smog, ground‐level ozone, acid rain (HNO_3_), and possibly global warming.^[^ ^1^, ^2^ ^]^ Since the turn of the millennium global NO_x_ emissions have, however, been leveling off^[^ ^1^ ^]^ and developed European countries^[^ ^3^ ^]^ have experienced a sharp decrease in NO_x_ emissions. This decrease is predominantly due to the use of abatement technologies and the transition away from fossil fuels (i.e., coal, oil, and natural gas) to renewable energies such as solar, wind, and hydropower. Due to the intermittent nature of most renewable energy sources^[^ ^4^, ^5^ ^]^ and the resulting impact on the required storage capacity size,^[^ ^6^ ^]^ a complete phase‐out of thermal electricity generation is improbable. Biomass‐fired power plants can help to stabilize the electrical grid without relying on fossil fuels, and their application has been on the rise in several regions.^[^ ^7^, ^8^, ^9^ ^]^ Nevertheless, firing of biomass can lead to accelerated deactivation of the catalyst for the selective catalytic reduction of NO_x_ with ammonia (NH_3_‐SCR), which is the preferred technology for NO_x_ removal,^[^ ^10^, ^11^, ^12^, ^13^ ^]^ due to a typically high content of alkali and alkali‐earth metals, especially potassium, in the flue gas. Aerosols containing alkaline potassium (often KCl or K_2_SO_4_) react strongly with acid sites of the catalyst, preventing NH_3_ surface adsorption, which is an essential step in the conversion process.^[^ ^14^ ^]^

Industrial NH_3_‐SCR catalysts for stationary applications (VWT catalysts) typically contain 1–5 wt.% V_2_O_5_ and 5‐10 wt.% WO_3_ or MoO_3_ supported on TiO_2_ (anatase) and can obtain > 90 % NO_x_ conversion if operated in a narrow and high temperature window of 300–400 °C.^[^ ^15^, ^16^ ^]^ One of several implantable strategies to make the catalyst more resistant to potassium is replacing the WO_3_ or MoO_3_ component by their respective Keggin‐structured heteropoly acids,^[^ ^17^, ^18^ ^]^ which also afford higher activity in the virgin state.^[^ ^19^ ^]^ The enhanced potassium tolerance is attributed to both an increased number of acid sites imparted by the heteropoly acid as well as more favorable redox properties. Incorporating vanadium into the Keggin structure, resulting in H_6_PV_3_Mo_9_O_40_ (HPA‐3) instead of using a mixture of H_3_PMo_12_O_40_ and V_2_O_5_ has also shown a slight promotional effect at temperatures between 250 and 350 °C.^[^ ^20^ ^]^ This improvement was primarily attributed to the enhanced redox window as measured by EPR spectroscopy. Furthermore, vanadium‐substituted Keggin structures provide more acid sites, which in turn are expected to boost their potassium tolerance.^[^ ^21^ ^]^ Thus, modifying pristine metal oxides such as WO_3_ and MoO_3_ to their corresponding Keggin‐type heteropoly acids has been reported as a promising strategy for improving the potassium tolerance, acidity of the catalyst, improving the redox activity, and enhancing the NO_x_ conversion and selectivity toward N_2_.^[^ ^22^, ^23^ ^]^ Placement of the SCR unit downstream of the dust‐removing unit is also demonstrated to slow down catalyst deactivation, due to lowered content of alkali‐metal‐containing aerosols,^[^ ^24^ ^]^ and tail‐end placement is expected to further lower the exposure to alkali metals. As the operating temperature of tail‐end SCR units is typically below 250 °C, development of novel, alkali‐resistant Keggin‐structured catalysts should focus on low temperatures to avoid excessive costs for gas reheating.

Several alternative transition metal‐based catalysts (Cr, Mn, Fe, Co, Cu), including Keggin‐type catalysts, have been reported to show good SCR activity down to about 250 °C.^[^ ^25^, ^26^, ^27^ ^]^ In all cases, effective NH_3_ adsorption, N‐H bond activation, and oxidation of NO to NO_2_ are important components for obtaining low‐temperature SCR performance. Also, a close coupling of redox properties and the surface acidic sites on the catalyst seems to be crucial for achieving excellent SCR activity and N_2_ selectivity in such catalysts.^[^ ^27^ ^]^ The Keggin structure of POMs has also been reported to act as the electron acceptor in certain catalysts.^[^ ^28^ ^]^ For example, Ke *et al*. revealed the electron redistribution between Ce in CeO_2_ and W in HSiW (H_4_SiW_12_O_40_), which increased the electron density of W in an HSiW/CeO_2_ catalyst and improved its N_2_ selectivity in the NH_3_‐SCR of NO_x_.^[^ ^29^ ^]^

Our study revealed the role of the cation‐anion interactions that influence the structure‐reactivity relationships in the POM catalyst and investigated the tolerance of TiO_2_‐supported HPA‐3 toward alkali metal contaminants, with a particular focus on the effects of potassium poisoning. The electron diffraction (ED) measurements investigated the arrangement of the various atoms in the supported HPA‐3 and its influence on the structure‐activity correlation. The impact of the exchange of counter cations, the synergistic effect between the redox‐active catalytic species, and the role of the presence of acidic sites on the activity of the supported HPA‐3 catalyst toward the NH_3_‐SCR of NO were all successfully investigated by EPR (Electron‐Paramagnetic Resonance) spectroscopy.

## 2 Materials and Methods

### 2.1 Chemicals

Molybdenum trioxide (99.5 %, Thermo Scientific), vanadium pentoxide (99.2 %, Alfa Aesar), hydrogen peroxide (30 %, VWR), phosphoric acid (85 %, Grüssing), potassium acetate (95 %, Grüssing), sodium acetate (99 %, Sigma Aldrich), cesium chloride (99 % +, Acros), and TiO_2_ (anatase, DT51, Tronox) were purchased from Cristal Global.

### 2.2 Synthesis of the HPA‐3 Catalyst

H_6_PV_3_Mo_9_O_40_ •3H_2_O was synthesized according to the literature using a two‐step method with nonsubstituted phosphomolybdic acid H_3_PMo_12_O_40_ precursor and the stable vanadium precursor H_9_PV_14_O_42_.^[^ ^30^ ^]^ H_3_PMo_12_O_40_ was obtained by heating MoO_3_ (0.620 mol) in an aqueous solution of H_3_PO_4_ (0.051 mol). H_9_PV_14_O_42_ was synthesized by dissolving water‐insoluble V_2_O_5_ (0.132 mol) in cold aqueous H_2_O_2_ (180 mL of 30 wt.% in water) followed by adding H_3_PO_4_ (0.020 mol). Subsequently, the aqueous vanadate precursor was added to the molybdate precursor solution under boiling conditions and heated for 30 minutes to obtain the product solution. Water was removed under vacuum to give a crystalline solid (yield > 95 %). The prepared materials were characterized by FTIR and compositional analysis using ICP‐OES. The K_5_HPV_3_Mo_9_O_40_, Na_6_PV_3_Mo_9_O_40_, and Cs_3.5_H_2.5_PV_3_Mo_9_O_40_ POM catalysts were prepared by the same procedure, and details are included in the Supporting Information.

### 2.3 Supported Catalyst Synthesis

In a typical synthesis, 925 mg of TiO_2_ anatase powder (DT51‐D, Tronox) was mixed with 75 mg of POM dissolved in 0.8 mL of deionized water, and the resulting slurry was kept at room temperature for a minimum of 1 hour prior to overnight drying at 90 °C resulting in 7.5 wt.% POM/TiO_2_. Tablets were formed by applying about 2.5 bars of pressure to the resulting material using a manual press, followed by crushing and sieving to the required particle size. Potassium‐loaded KPV‐3/TiO_2_ (35 and 100 εmolK/g_cat_) was prepared by impregnating the catalysts with KNO_3_ dissolved in deionized water, followed by overnight drying at 90 °C and calcining at 400 °C in air (2 hours, 100 °C/hour) to decompose KNO_3_.

### 2.4 Catalyst Characterization

X‐ray powder diffraction (XRD) measurements were carried out on a Huber G670 powder diffractometer using Cu Kα radiation within a 2θ range of 5–85° in steps of 0.005° at a speed of 0.02 s^−1^.

Inductively coupled plasma optical emission spectroscopy (ICP‐OES) was used to determine the compositional analysis of the bulk POMs and the weight percentage of Mo and V contained in the impregnated catalysts. The measurements were carried out using a Perkin Elmer Plasma 400 by dissolving ca. 20 mg of the HPA‐n catalyst in 250 mL of double distilled water. For evaluation of the composition of the supported catalysts, sample preparation was done by carefully dissolving the catalysts in aqua regia (3:1 mixture of concentrated HNO_3_ and HCl) in a microwave oven. Phosphorous, molybdenum, and vanadium ICP‐standard solutions (1000 µg/mL) were used for calibration.

Both freshly synthesized HPA‐n materials and supported HPA‐n catalysts were analyzed by Fourier‐transformed infrared spectroscopy (FTIR) at ambient conditions using a Jasco FT/IR‐4100 spectrometer equipped with a PIKE GladiATR ATR‐accessory with a resolution of 4 cm^−1^. The FTIR spectra were recorded in the range between 4000 and 500 cm^−1^.

Ammonia temperature‐programmed desorption (NH_3_‐TPD) experiments were conducted on a Micromeritics Autochem‐II instrument equipped with a TCD detector. 100 mg of sample was placed in a quartz tube and pretreated in helium flow at 100 °C for 1 hour. The sample was then treated with anhydrous NH_3_ gas (1 vol.% NH_3_ in He) at 100 °C. After NH_3_ adsorption, the sample was treated with helium (50 mL/minute) for 1 hour at the same temperature to remove loosely bound NH_3_. Finally, the desorption was carried out by heating the sample from 100 to 420 °C (10 °C/minute) with 15 minutes dwelling at 420 °C under a flow of helium (50 mL/minute). Both the dynamic‐ and static‐temperature sections were used for the determination of the relative number of acid sites.

H_2_‐temperature programmed reduction (H_2_‐TPR) experiments were also conducted on a Micromeritics Autochem‐II instrument equipped with a TCD detector. 100 mg of sample was placed in one arm of a U‐shaped quartz sample tube on a quartz wool plug. The TPR analysis was carried out in a reducing mixture (50 mL/minute) consisting of 1 % H_2_ and balance Ar from room temperature to 420 °C (10 °C/minute ramping) with 15 minutes dwelling at 420 °C.

Dynamic thermogravimetric analysis (TGA) experiments were conducted on a Mettler Toledo (TGA/DSC1 STARe System) instrument. In a typical experiment, 40 mg of sample was heated from room temperature to about 410 °C at a rate of 10 °C/minute under an N_2_ flow (50 mL/minute).

X‐ray photoelectron spectroscopy (XPS) analyses were performed using a Thermo Scientific system at room temperature using Al Kα radiation (1484.6 eV) and a spot size of 400 µm. The spectra of Mo3d, Ti2p, V2p_3/2_, Na1s, K2p, and Cs3d were measured with a pass energy of 50 eV and a step size of 0.1 eV. A flood gun was used to reduce sample charging effects, and the obtained spectra were further corrected by setting the C1s binding energy at 284.8 eV. Data processing was done using the Avantage 4.87 software. The area under the curves using smart backgrounds was correlated to the relative elemental concentrations using the following ALOTHERMO1 sensitivity factors: Mo3d (11.008), Ti2p (6.741), V2p_3/2_ (6.118), Na1s (10.588), K2p (4.671), and Cs3d (49.629).

Continuous rotation 3D electron diffraction (3D‐ED) data for H‐POM (H_6_[PMo_9_V_3_O_40_]•3(H_2_O)) and K‐POM (K_5_H[PMo_12_O_40_]•7(H_2_O)) were collected using a Rigaku XtaLAB Synergy‐ED dedicated electron diffractometer, equipped with a Rigaku Oxford Diffraction HyPix detector.^[^ ^31^, ^32^ ^]^ Details on the data collection are compiled in the Supporting Information.

EPR measurements were conducted on a CW X‐band Bruker EMX spectrometer equipped with an ER 4102ST cavity. Ex‐situ experiments were performed on weighted amounts of samples measured in 4 mm EPR tubes in the same experimental campaign as a series of 5 reference samples consisting of a solid solution of VOSO_4_⋅3H_2_O in K_2_SO_4_ with vanadium content verified by elemental analysis (ICP). All in‐situ measurements were conducted in flow‐through quartz tubes using quartz wool to position the sample in a plug flow reactor setup. 20–30 mg of sample (particle sizes 180–300 µm) was loaded, and the reactor was inserted within the resonator. All of the sample was within the measured area. Temperature control was obtained by connecting the reactor to a Bruker EMX variable temperature heater regulated by a thermocouple above the sample, and monitored by a thermocouple below the sample. The gas flow was controlled by MKS and Bronkhorst mass flow controllers on two separate gas lines heated at 80 °C to avoid condensation. Measurements were performed at a microwave power of 6.6 mW, frequency of 9.457 GHz, modulation amplitude of 5.2 G, modulation frequency of 100 kHz, field range of 240 mT to 420 mT. The time resolution was 1 spectrum each 69 seconds. All spectra were background corrected using the fully oxidized spectrum of the catalyst as well as linear background correction. Intensities of spectra were obtained by simulating the individual spectra and then performing double integration on the simulated spectra. This was done since the low vanadium loading makes the procedure of direct integration of experimental spectra very vulnerable to fluctuating baseline effects, which is unavoidable when measuring at 250 °C. The simulations were based on the spin Hamiltonian parameters given in Table S12. Intensities at temperatures above room temperature (295 K) were corrected using the Boltzmann equation, which in this case simplifies to *T*/(295 K) where *T* is the temperature in K, and were correlated to the vanadium content by comparison of the room temperature spectrum of each sample with the spectrum of the same sample measured during the ex‐situ quantification experiment. Data treatment was performed using Matlab 2023b with EasySpin 6.0.6,^[^ ^33^ ^]^ on all spectra in a truncated range from 260 to 400 mT. Spectra and intensity profiles have undergone light smoothening using a Savitzky‐Golay method based on second‐order polynomials. Further details of the measurements can be found in the Supporting Information.

### 2.5 Catalyst Activity Measurements

NH_3_‐SCR activity measurements were performed on 50 mg of catalyst (35–45 mesh, 355–500 µm) in a tubular reactor (inner diameter 3.7 mm) using a feed gas containing 600 ppm NO, 600 ppm NH_3_, 4.5 vol. % O_2_, and ca. 2 vol. % H_2_O with N_2_ as balance. The concentrations of NO, NO_2_, NH_3_, and N_2_O from the reactor outlet were measured by an Antaris IGS flue gas analyzer (Thermo Scientific, USA) and compared to measurements on the gas stream bypassing the reactor. Only steady‐state data at each temperature was considered, and the testing was performed in ascending temperature order. The NO_x_ and NH_3_ conversions were calculated using their respective inlet and outlet concentrations as given by Equations (1), (2) and (3). The first‐order rate constant was calculated with Equation (4) assuming plug flow conditions and first‐order reaction with respect to NO, where F was the total flow rate (NmL/s), w the catalyst or vanadium mass (g), and $X_{NO_{x}}$ the fractional NO_x_ conversion.

(1) $$\left[NO_{x}\right]=\left[NO_{2}\right]+\left[NO\right]$$

(2) $$X_{NO_{x}}\left(\%\right)=\frac{{\left[NO_{x}\right]}_{in}-{\left[NO_{x}\right]}_{out}}{{\left[NO_{x}\right]}_{in}}\times 100\;\%$$

(3) $$X_{NH_{3}}\left(\%\right)=\frac{{\left[NH_{3}\right]}_{in}-{\left[NH_{3}\right]}_{out}}{{\left[NH_{3}\right]}_{in}}\times 100\;\%$$

(4) $$k=-\frac{F}{w}ln\left(1-X_{NO_{x}}\right)$$

## 3 Results and Discussion

### 3.1 Catalyst Characterization

Initially, the Keggin‐type POMs H_6_PV_3_Mo_9_O_40_, Na_6_PV_3_Mo_9_O_40_, K_5_HPV_3_Mo_9_O_40_, and Cs_3.5_H_2.5_PV_3_Mo_9_O_40_, were synthesized and characterized by elemental analysis (ICP‐OES, Tables S1‐S4), as well as TGA and FTIR spectroscopy (Figures S1‐S4). XRD was also conducted on the pure POM materials to determine their phase purity (Figure S5). The reflections at 2Ө of 8.1° and 9.5° in the XRD profiles confirmed the presence of the Keggin anion in all the catalysts,^[^ ^34^ ^]^ and the Cs‐POM showed sharp reflections compared to the other POM catalysts. Moreover, FTIR for the pure POM catalysts showed characteristic infrared bands between 600 and 1100 cm^−1^ as shown in Figures S1‐S4. These bands match the distinctive stretching vibrations for the Keggin oxo‐anions of P‐O_a_, Mo = O_d_, Mo‐O_b_‐Mo, and M‐O_b_‐Mo bonds that were detected at 1045, 948, 869, and 739 cm^−1^, respectively, where O_b_ refers to the oxygen atom that connects the two trimetallic groups, O_c_ joins the two octahedral MoO_6_ units inside the trimetallic group, O_d_ is the terminal oxygen atom, and O_a_ is the oxygen atom of the PO_4_ unit of the tetrahedron and trimetallic group Mo_3_O_13_. Vanadium was successfully incorporated into the Keggin structure of the POM catalysts, as evidenced by the shift (drop) in the IR bands when compared to the nonsubstituted parent heteropoly acid.

In the case of the cation‐exchanged POMs supported on TiO_2_ (anatase), denominated in the following as XPV_3_/TiO_2_ (X = H, Na, K, Cs), the band for the P‐O_a_ stretching was shifted from 1045 to 1054 cm^−1^, the band for Mo = O_d_ shifted from 948 cm^−1^ to 964 and 960 cm^−1^ for CsPV‐3/TiO_2_ and HPV_3_/TiO_2_, respectively, and to 959 and 954 cm^−1^ for KPV_3_/TiO_2_ and NaPV_3_/TiO_2_, respectively. Whereas, in the region 850–550 cm^−1^ a very broad band was observed, which corresponded to the presence of the Ti‐O stretching vibrations of the support (500–640 cm^−1^) and (in the region below 800 cm^−1^ (up to 650 cm^−1^)) the vibrational bands of the metal–metal bridging oxygen–metal bonds can be observed for the edge M–O–M linked octahedrons (870 cm^−1^ for Mo‐O_b_‐Mo and 740 cm^−1^ for V‐O_b_‐Mo). This low intensity of the bands may be due to the low concentration of the POM on the support.

The vanadyl species showed a broad peak (V‐O_b_‐Mo) due to their low concentration as compared to the support and the promoter species. The retention of the characteristic peaks in the supported catalysts indicates that the Keggin structure of the POMs remains intact after impregnation. Thus, the shift in the bands in the FTIR spectra at higher frequency after the cationic exchange of the supported catalyst may be due to the coordination of the POM to the respective cations or cationic‐anionic interactions.

**Figure 1:**
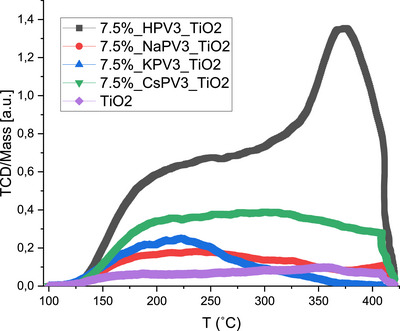
Temperature‐programmed NH_3_ desorption (NH_3_‐TPD) profiles of the 7.5 wt.% XPV_3_/TiO_2_ (X = H, Na, K, Cs) catalysts and neat TiO_2_ as a benchmark.

Figure 1 displays the NH_3_‐TPD profiles (100–450 °C) measured for the supported POM‐catalysts. NH_3_‐TPD was used to determine the surface acidity of the various synthesized POM catalysts, which likely affected the SCR activity. The total amount of adsorbed ammonia, which is determined from the area under the TPD curve, corresponds to weakly adsorbed ammonia (desorption temperature around 50–150 °C), moderate acidic sites around 150–300 °C and strongly adsorbed ammonia (desorption temperature above 300 °C). All the catalysts showed a broad and intense peak in the region of 250–425 °C revealing the presence of moderate to strong acidic sites. These sites originate both from the TiO_2_ support and the vanadium present in the catalyst.^[^ ^20^ ^]^ The 7.5 wt.% HPV_3_/TiO_2_ catalyst showed the highest acidity, exhibiting two desorption peaks, whereas the peak intensity of the cation‐exchanged POMs, where the protons in the POM were exchanged by Na, K, or Cs ions, was considerably lower (as expected), with the KPV_3_/TiO_2_ catalyst appearing to have less strong acid sites compared to NaPV_3_/TiO_2_ and CsPV_3_/TiO_2_. The trend of acidity was therefore found to be HPV_3_/TiO_2_ > CsPV_3_/TiO_2_ > NaPV_3_/TiO_2 _> KPV_3_/TiO_2_. The relative number of the acidic sites measured by the NH_3_‐TPD analysis is shown in Table S5 together with an elaborated discussion. Thus, in agreement with the FTIR analysis, where a slight shift of the bands was observed, the cation exchange influenced both the structure of the POMs and lowered their total acid strength.

Another interesting feature could be deduced from the measured TGA profiles of the anatase‐supported POMs (Figure S6). Here, CsPV_3_/TiO_2_ showed a significantly higher water desorption temperature peak at around 240 °C compared to the other three POM materials, which all had a temperature peak centered around 170 °C. Moreover, a second temperature peak at around 280 °C appeared for KPV_3_/TiO_2_, suggesting that water molecules with different bonding modes and strengths could be present in the different materials.

**Figure 2:**
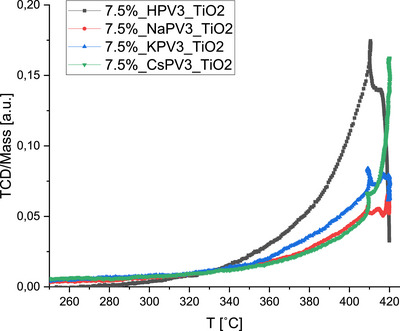
Temperature‐programmed reduction (H_2_‐TPR) profiles of the 7.5 wt.% XPV_3_/TiO_2_ (X = H, Na, K, Cs) catalysts.

To study the redox properties of the various POM catalysts, H_2_‐TPR measurements were performed. The TPR profiles of all 7.5 wt.% XHPV_3_/TiO_2_ (X = Na, K, Cs) catalysts are shown in Figure 2. The catalysts showed a reduction peak between 300 and 425 °C (assigned to the reduction of V^5+^ to V^4+^).^[^ ^18^ ^]^ The slight difference in reduction temperatures for the catalysts is likely due to the combined effect of the vanadium content, promoter formulations, and constituent elements/cations associated with the HPAs.

**Figure 3:**
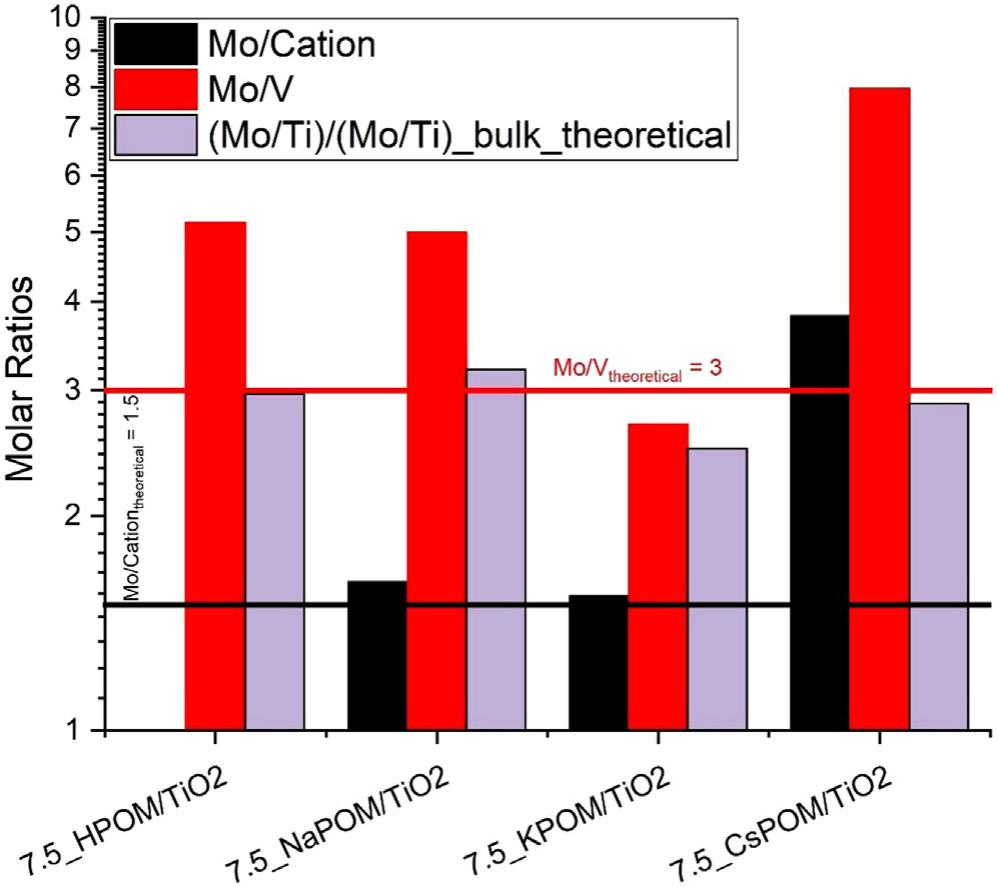
Surface concentration ratios of the 7.5 wt.% XPV_3_/TiO_2_ (X = H, Na, K, Cs) catalysts as determined by XPS.

The relative surface concentrations of the respective elements in the 7.5 wt.% XHPV_3_/TiO_2_ (X = Na, K, Cs) catalysts were next measured by XPS (Figure 3). The Na‐ and K‐containing catalysts exhibit molar Mo to cation ratios very close to the theoretical value of 1.5, corroborating the elemental bulk analysis of the neat POM materials by ICP‐OES (Tables S2 and S3). On the other hand, the 7.5 wt.% CsHPV_3_/TiO_2_ catalyst showed a significantly higher Mo/Cs ratio, in line with its lower Cs content as measured by ICP‐OES analysis (Table S4). The measured surface Mo/Ti molar ratios are between 2.5 and 3.2 times higher than the corresponding values based on the theoretical bulk concentrations. The narrow range of the surface enrichment factor indicates a similar degree of POM dispersion on the TiO_2_ surface. The significant surface enrichment rules out that a substantial share of POMs is present in big agglomerates sized larger than the penetration depth of the used X‐rays of a few nm.

**Figure 4:**
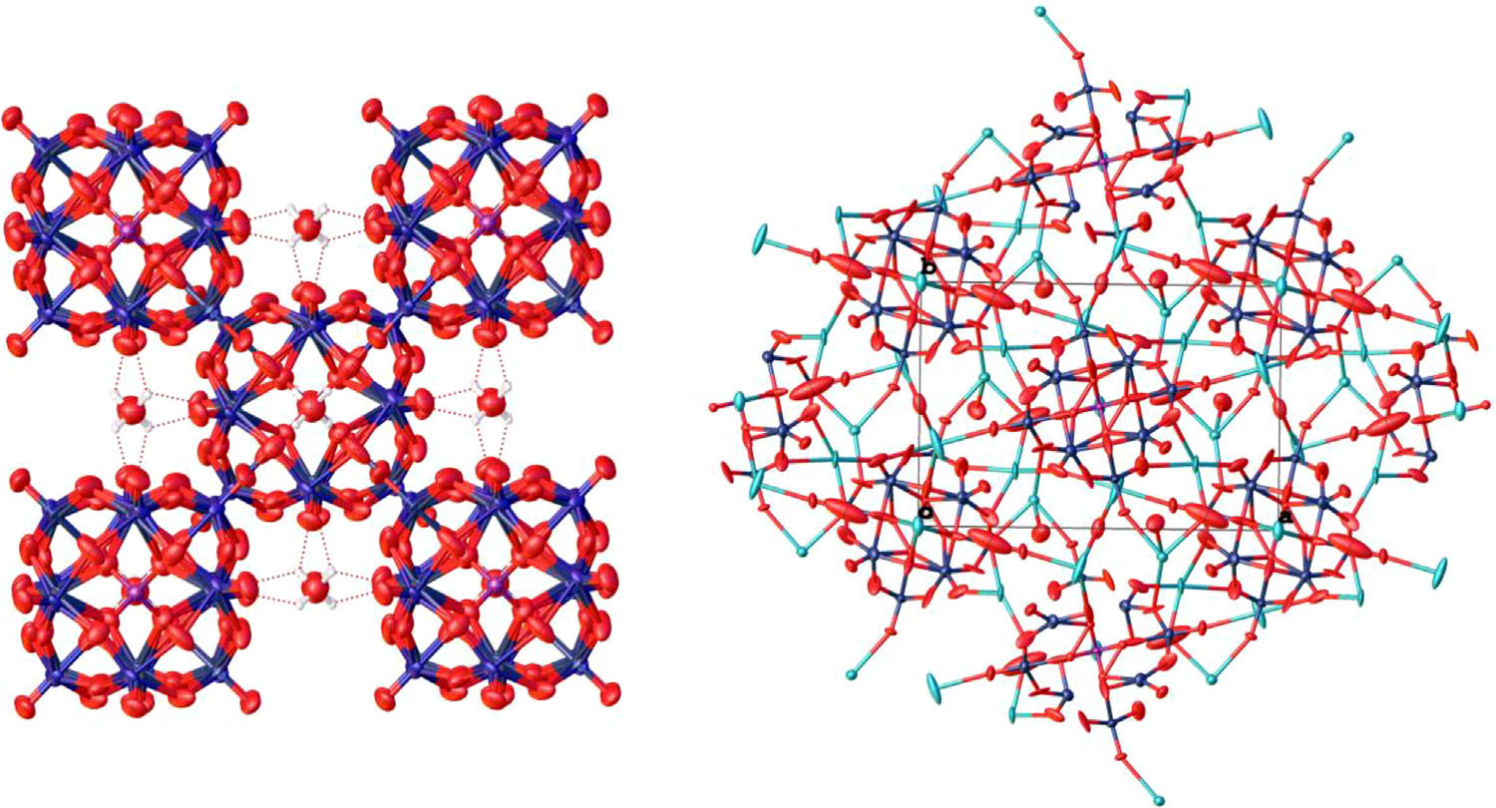
Solid‐state structures of H‐PV_3_ (left) and K‐PV_3_ (right), as determined by electron diffraction. Red: O, dark blue: Mo, V, purple: P, cyan: K., CCDC deposition numbers: 2 492 701, 2 453 880 contain the supplementary crystallographic data for this paper. These data are provided free of charge by the joint Cambridge Crystallographic Data Centre and Fachinformationszentrum Karlsruhe (http://www.ccdc.cam.ac.uk/structures).

The crystal structures of H‐POM and K‐POM (Figure 4) (H atoms not included in the structural model) were determined by applying continuous rotation 3D electron diffraction (3D‐ED) analysis. Single‐crystal diffraction data were sufficient for the analysis of the structure (Tables S6‐S11)

To explain the significant differences in the catalytic performance observed for the POM salts with different cations, knowledge of the cation‐anion interaction in these POM salts is required, as shown in Figure 4 and Tables S6‐S7. Unfortunately, POMs with hard cations (H^+^, Na^+^) are difficult to crystallize due to their high solubility in water, in contrast growing single crystals from POM salts with soft cations (Cs^+^) is impossible due to their low solubility. Instead of single crystal x‐ray diffraction (SCXRD), we therefore employed continuous rotation 3D electron diffraction (ED) to obtain structural information from microcrystalline powder. We were able to successfully elucidate solid‐state structures of the H‐POM and K‐POM (Figure 4), which we compared to the previously published structure of Na_4_H_2_PV_3_Mo_9_O_40_ (determined by SCXRD, CCDC deposition number 2 205 007).^[^ ^35^ ^]^ H‐POM crystallized in the cubic space group I‐43 m, whereas Na_4_H_2_PV_3_Mo_9_O_40_ crystallized in the tetragonal space group P‐42_1_c, and K‐POM in the orthorhombic space group Pnnm. All three compounds feature unit cells containing 2 POM anions. Both, the H‐POM and the K‐POM feature a body‐centered packing of the POM anions. In the structure of H‐POM the metal site was modeled as Mo (75 % atomic occupancy) and V (25 % occupancy), and the locations of the metals were freely refined. The six protons per formula unit, which are presumed to balance the charge of the [PMo_9_V_3_O_40_]^6−^ anion could not be located. In the structure of K‐POM due to difficulties in the determination of the accurate orientation of hydrogen bonds, hydrogen atoms of water molecules were not included in the structural model.

In case of the H‐POM, three O atoms per formula unit were found between the POM anions, which could be interpreted as H_2_O or as H_3_O^+^. To compensate for the charge a total of six protons is required, therefore, we can conclude, that at least three protons must be bound to the POM anions. The distance of the POM anion (O4) to the free O is 3.27 Å. The closest distance between two POM anions (O3 to O4) in case of the H‐POM is 2.68 Å. This is consistent with a covalently bound H atom with a hydrogen bond to the next POM anion. In the structure of Na_4_H_2_PV_3_Mo_9_O_40_, the distances between the POM anion and the Na^+^ cations have been reported as 3.00 Å, indicating a predominantly ionic interaction not a covalent bond. In contrast, the distances between the POM anion and the K^+^ cation in the structure of K‐POM range between 2.63 Å and 2.86 Å (sum of covalent radii of O and K is 2.59 Å),^[^ ^36^ ^]^ which is significantly shorter despite the larger radius of K (vs. Na). This indicates a stronger interaction between the cation and the anion with heavier alkali elements. Elemental analysis by ICP‐OES indicated the presence of 5 K atoms per POM, whereby 6 K atoms per POM anion were found in the solid‐state structure. Structural disorder and the true chemical composition resulted in not full site occupancy of some potassium atoms. Their occupancy was freely refined, leading to an average number of 5.4 K atoms per POM, which indicates the presence of one covalently bound H atom. Since the framework elements V and Mo are indistinguishably distributed over all metal sites (not separately modeled for K‐POM due to insufficient data quality), it is not possible to discern whether there is a preferential interaction of the cations with oxo ligands bound to V or to Mo. However, based on the catalytic results it is safe to conclude, that the stronger interaction of the cations with the POM inhibits catalytic activity. This seems not to be the case for the protons, which leads to the conclusion, that they must play an active role in the catalytic mechanism. This is in line with the observation, that the H‐POM performed best in the catalytic experiments.

### 3.2 NH_3_‐SCR Activity

**Figure 5:**
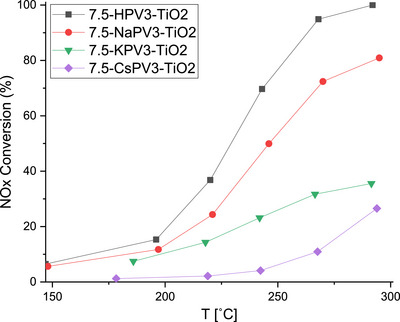
NO conversion during NH_3_‐SCR with 7.5 wt.% XPV_3_/TiO_2_ (X = H, Na, K, Cs) catalysts. Reaction conditions: [NO] = [NH_3_] = 600 ppm, [O_2_] = 4.5 vol %, balanced by N_2_, WHSV = 240000 mL/g·h.

After characterization of the neat and the supported POM catalysts, the 7.5 wt.% XPV3/TiO_2_ (X = H, Na, K, Cs) catalysts were evaluated for their catalytic activity in NH_3_‐SCR (Figure 5). The catalytic activity decreased with increasing basicity of the counter‐cation in the entire temperature range. This is in line with former reports on the deactivating power of alkali and alkaline‐earth metals.^[^ ^12^, ^13^, ^14^ ^]^ Apparent activation energies (*E*_a_) in the temperature range of 215–275 °C were derived from Arrhenius plots in Table 1 and Figure S7. The decrease in *E*_a_ when exchanging H for Na‐ or K‐cations is well known from literature^[^ ^15^, ^16^ ^]^ and reflects the fact that alkali metals reduce the number and strength of the acid sites on the surface, which is detrimental to the required adsorption of NH_3_. The reduced adsorption of NH_3_ becomes more of a bottleneck at higher temperatures, hence the lower E_a_. Like *E*_a_, the pre‐exponential factor *A* also decreases markedly going from H via Na to K as counter cation. Cs as counteraction resulted in an unexpectedly high *E*_a_ of 75.9 kJ/mol which is in between the H‐ (86.3) and Na‐containing (69.8) catalysts. Also, its pre‐exponential factor does not follow the trend seen between H and K. Its relatively high *E*_a_ can be explained by its extremely low activity at temperatures below 250 °C, probably caused by limitations in its redox properties as shown by EPR spectroscopy (see below).

**Table 1:** Kinetic parameters for NH_3_‐SCR using XPV_3_/TiO_2_ (X = H, Na, K, Cs) catalysts.

Catalyst	*E*_a_^[^ ^a^ ^]^ [kJ/mol]	*A* ^[^ ^b^ ^]^ [mL/g⋅s]	*k*_250C_^[^ ^c^ ^]^ [mL/(g⋅s]	Redox active V sites according to EPR [wt% V]
7.5 wt.% HPV_3_/TiO_2_	86.3	4.33⋅10^10^	103	0.23
7.5 wt.% NaPV_3_/TiO_2_	69.8	4.55⋅10^8^	49	0.22
7.5 wt.% KPV_3_/TiO_2_	41.0	2.40⋅10^5^	20	0.13
7.5 wt.% CsPV_3_/TiO_2_	75.9	1.56⋅10^8^	4	∼0

^[a]^ Obtained by Arrhenius plots using NO conversions at 215‐275 °C shown in Figure 6.

^[b]^ Pre‐exponential factors.

^[c]^ Obtained by interpolation using *E*_a_ and *A*.

The interpolated first‐order rate constant at 250 °C (*k*_250C_) of 7.5 wt.% HPV_3_/TiO_2_ of 103 mL/g⋅s is close to the relatively high value previously found for a 3 wt.% V_2_O_5_‐15 wt.% H_3_PMo_12_O_40_/TiO_2_ catalyst (116 mL/g⋅s)^[^ ^23^ ^]^ measured under similar conditions. Importantly, no N_2_O formation was detected with the supported catalyst, meaning that the N_2_ selectivity was 100 % within the entire temperature range for all the catalysts.

**Figure 6:**
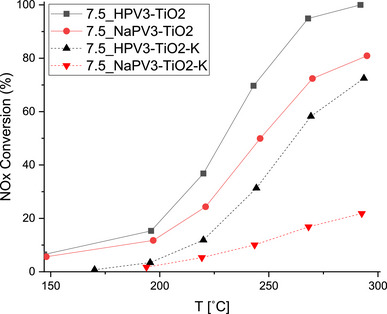
NO conversion during NH_3_‐SCR of fresh and K‐poisoned (100 µmolK/g_cat_) 7.5 wt.% XPV_3_/TiO_2_ (X = H, Na) catalysts. Reaction conditions: [NO] = [NH_3_] = 600 ppm, [O_2_] = 4.5 vol. %, balanced by N_2_, WHSV = 240,000 mL/g·h.

From an application point of view, the hydrogen‐containing HPV_3_/TiO_2_ catalyst seems—based on the measured NH_3_‐SCR activities and its easier synthesis—the most promising catalyst. However, if to be used in biomass‐fired power plants, the catalyst should exhibit good tolerance toward alkali metals, chiefly potassium. As the 7.5 wt.% NaPV_3_/TiO_2_ catalyst at 250 °C was only about 50 % less active than the analogous HPV_3_/TiO_2_ catalyst, and the presence of Na might protect it from further deactivation by potassium, this catalyst was also tested for K‐poisoning. Figure 6 shows the effect of 100 εmolK/g_cat_ on the activities of the two 7.5 wt.% XPV_3_/TiO_2_ catalysts (X = H, Na). Obviously, both catalysts were severely deactivated by potassium, and the presence of Na countercations did not protect against further poisoning by K. The interpolated *k*_250C_ for the 7.5 wt.% HPV_3_/TiO_2_‐K and 7.5 wt.% NaPV_3_/TiO_2_‐K catalysts are 33 and 8 mL/g⋅s corresponding to relative activity losses of 68 % (H‐form) and 84 % (Na‐form), respectively (see Table 2 and Figure S8). Notably, the K‐poisoned catalysts did not produce N_2_O and showed no substantial overconsumption of NH_3_ (Figure S9).

**Table 2:** Comparison of NH_3_‐SCR activities at 250 °C of fresh and K‐loaded 7.5 wt.% XPV_3_/TiO_2_ (X = H, Na) catalysts.

Catalyst	K loading [µmolK/g_cat_]	K/V [mol/mol]	*k*_250C_^[^ ^a^ ^]^ [mL/g⋅s]	*k*_250C_K_/*k*_250C_fresh_
7.5 wt.% HPV_3_/TiO_2_	‐	‐	103	‐
7.5 wt.% HPV_3_/TiO_2_	100	0.87	33	0.32
7.5 wt.% HPV_3_/TiO_2_	35	0.31	51	0.50
7.5 wt.% NaPV_3_/TiO_2_	‐	‐	49	‐
7.5 wt.% NaPV_3_/TiO_2_	100	0.93	8	0.16

^[a]^ Obtained by Arrhenius plots and interpolation using *E*_a_ and *A*.

The severe activity losses stand in contrast to the ones previously observed for 15 wt.% H_3_PMo_12_O_40_/TiO_2_ catalyst doped with 3 or 5 wt.% V_2_O_5_,^[^ ^14^ ^]^ which experienced much milder deactivation by 100 εmolK/g_cat_. Loading the 7.5 wt.% HPV_3_/TiO_2_ catalyst with 35 εmolK/g_cat_ (K/V ratio of 0.305), allowed better comparison to the previous study^[^ ^14^ ^]^ and led to less deactivation of only 50 % at 250 °C. However, this value is still considerably higher than experienced by 3 wt.% V_2_O_5_‐15 wt.% H_3_PMo_12_O_40_/TiO_2_ (22 %) at the same K/V molar ratio. Hence, incorporating vanadium into the Keggin‐structure of phosphomolybdic acid does not afford NH_3_‐SCR catalysts with high tolerance toward potassium as it is desired in biomass‐fired power plants.

### 3.3 EPR Measurements

**Figure 7:**
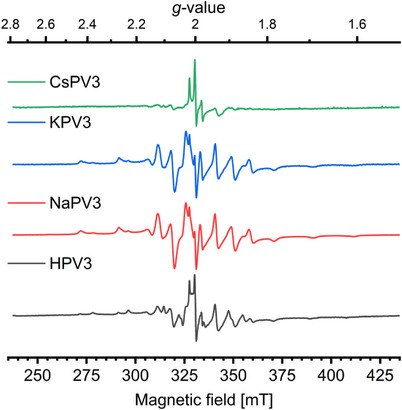
Ex‐situ EPR spectra (spectra are plotted in arbitrary units normalized to sample mass) of fresh samples obtained during quantification experiments at 77 K. The features correspond to oxidovanadium(IV) species.

EPR has previously been successful for correlating catalytic activity with the amount of redox‐active vanadium in POM structures.^[^ ^20^ ^]^ The same type of investigation was performed on the series of samples from Table 1. Of the two relevant oxidation states of vanadium, V(IV) is paramagnetic and gives an informative EPR signal, whereas V(V) is diamagnetic and EPR silent. The molybdenum in the POM structures is always Mo(VI), which is diamagnetic and does not influence the EPR investigation. The EPR investigation consisted of two separate experiments: 1) An ex‐situ quantification experiment of the V(IV) content of all fresh samples at 77 K to calibrate the signal intensity versus a series of external reference samples, see Figure 7. 2) In‐situ experiments where the EPR signal of the entire plug flow reactor was followed while the sample was exposed to the reaction mixture. The experimental protocol for the in‐situ experiments was designed to start in the fresh state at room temperature and to proceed to probe and quantify both the fully reduced and the fully oxidized states of each sample by cycling between a reducing gas flow (NO + NH_3_) and an oxidizing gas flow (NO + O_2_) at 250 °C.^[^ ^37^ ^]^ The fully oxidized state of the catalysts was reproducible and essentially EPR silent with only a few weak features. Since good background corrections are crucial for reliable quantification of EPR signal intensity, the fully oxidized spectrum obtained at 250 °C was used as background for all other spectra at this temperature.

The in‐situ experimental protocol consisted of the following steps: The total flow was kept at 200 NmL/minute, corresponding to a GHSV of approximately 200,000 h^−1^. Between Steps 2 and 6, a flush with He gas for 10 minutes was performed.

1. The fresh catalyst was measured at room temperature.
2. Flow of 10 % O_2_ in He was applied, and the temperature was ramped to 250 °C by 10 °C/minute. The sample was left at this temperature for 30 minutes.
3. Reduction: Flow of 500 ppm NO and 550 ppm NH_3_ in He at 250 °C.
4. Oxidation: Flow of 500 ppm NO and 4.5 % O_2_ in He at 250 °C.
5. Reduction (repetition of Step 3).
6. SCR conditions: Flow of 500 ppm NO, 550 ppm NH_3_ and 4.5 % O_2_ in He at 250 °C.
7. Reduction (repetition of Step 3).

We obtained two types of results from the EPR investigation: 1) The individual spectra, containing information about the coordination of vanadium (IV), and 2) the quantification of the EPR signal intensity of vanadium(IV). The latter is the most important in this investigation, since it reveals the amount of vanadium in the sample, which is interacting with the reaction gases. Only vanadium that was able to change oxidation state when the gas was switched between the two gas mixtures can engage in the SCR reaction.^[^ ^37^ ^]^

**Figure 8:**
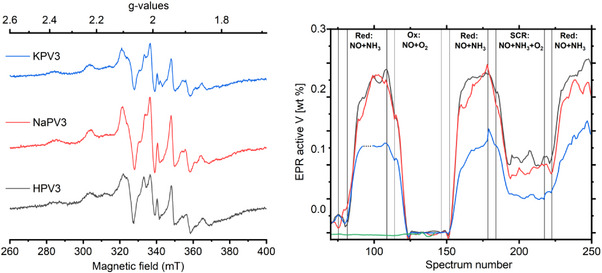
**Left**: In‐situ EPR spectra (523 K, spectra are plotted in arbitrary units normalized to sample mass) obtained during the reduction (NO + NH_3_). The features correspond to oxidovanadium(IV) species but are broader than in Figure 7. **Right**: EPR active vanadium during Steps 3–7 of the in situ protocol of HPV_3_ (black), NaPV_3_ (red), KPV_3_ (blue), and CsPV_3_ (green). He flushes were performed in the time period between close vertical lines. To match the timeline of switches between gas mixtures, an artificial gap marked as a dotted line segment was added for KPV_3_ around spectrum number 96, and 10 spectra around spectrum 180 were cut out. The experiment for CsPV_3_ was discontinued after the first reduction and oxidation due to lack of detectable signal.

The individual EPR spectra obtained during the quantification experiments, see Figure 7 and during the in‐situ experiments, see Figure 8 left were all similar, with small differences due to different resolution. They correspond to known literature spectra of oxidovanadium(IV) (vanadyl) species with an apical oxido group and a basal plane of 4 oxygen donor atoms. The rich fine structure in the spectra is due to the interaction with the nuclear spin of vanadium (100 % of isotope ^51^ V with *I* = 7/2) and the presence of axial anisotropy. The spectra are well resolved during measurements at low temperature, see Figure 7. The spectra were modeled using the same 3 species as used in previous investigations,^[^ ^20^ ^]^ see the characteristic spin Hamiltonian parameters in Table S12: Two subtly different vanadium species with an axial type of spectrum and a contribution with a broad spectrum. The broadening is either due to interaction with other paramagnetic centers or due to variations in the coordination environment. At reaction conditions, the spectra were less resolved due to the high temperature, which decreased the signal‐to‐noise ratio, and some of the weaker outer satellites in the spectra were lost in the background, see Figure 8 left for selected spectra. The spectral features did not really depend on the counter ion for H^+^, Na^+^, and K^+^. The Cs^+^‐exchanged sample was different since it was essentially nonreducible at the applied reaction conditions. The EPR spectrum of this sample was essentially below the detection limit at in‐situ conditions both in the reduced and oxidized states and the experiment was cut short.

The EPR intensity profile as a function of time during the in‐situ protocol is shown in Figure 8 right. The y‐axis was recalculated from EPR signal intensity (obtained by double integration of individual EPR spectra) to content of vanadium via intensity of reference spectra and the Boltzmann distribution as described in the Experimental Section. The intensity profiles show that 1) vanadium(V) was reduced to vanadium (IV) on HPV_3_, NaPV_3_ and KPV_3_, but not on CsPV_3_, 2) the subsequent oxidation reoxidized all reduced vanadium back to vanadium(V), 3) the reduction was completely reproducible, and 4) the samples all had an intermediate state during SCR conditions, probably due to a gradient of reduced and oxidized vanadium throughout the catalyst sample during catalysis. The quantification of the results showed that all vanadium was vanadium(V) when the sample was in the O_2_ gas in step 2 of the experimental protocol and in the NO + O_2_ gas mixture in step 4. Approximately 0.23 wt.% vanadium was reduced to V(IV) on the HPV_3_ sample and 0.22 % vanadium on the NaPV_3_ sample. The total amount of vanadium on the sample was 0.68 wt.% for HPV_3_ and 0.63 wt.% for NaPV_3_. Thus, the amount of reducible vanadium corresponded approximately to one vanadium out of three per POM unit in these two samples. On KPV_3_, the value was lower at around 0.13 wt.% reducible vanadium out of 0.60 wt.% total vanadium on the sample. The corresponding experiment for CsPV_3_ showed only a negligible amount of reduced vanadium during the first reduction step. The values are collected in the right column of Table 1.

The amount of reducible vanadium was severely influenced by the counter ion. The quantification by EPR revealed only a small difference between the available redox‐active vanadium in the HPV_3_ and NaPV_3_ samples whereas, the catalytic activity measured as the rate coefficient was decreased by approximately 50 %. This shows that the number of redox active vanadium sites was not the only factor. When comparing the NaPV_3_, KPV_3_ and CsPV_3_, the catalytic activity correlated directly with the quantification of redox‐active vanadium. The main difference between the alkali‐exchanged samples on one hand and the HPV_3_ sample on the other hand is the acidity, see Figure 1. HPV_3_ has a large contribution, whereas the other three were similar. We suggest that the SCR activity of the different POMs supported on TiO_2_ was a combination of both the reducibility of vanadium sites and the Brønsted acidity. Sodium exchange deactivated the sample by removing the highly acidic sites, but the overall reducibility was not decreased. The larger alkali metal cations K^+^ and Cs^+^ remove the acidity in the same way as Na^+^, but in addition they also decrease the ability to be reduced by NO + NH_3_. This is more detrimental to catalytic activity than blocking the acidic sites. Na^+^, K^+^, and Cs^+^ are chemically very similar, but their sizes are very different. The strong dependence on the identity of the cation indicates that the cation is physically blocking the access of NO to the redox‐active metal.

## 4 Conclusion

Various cation‐exchanged (H/Na/K/Cs) vanadium‐substituted polyoxometalate catalysts supported on TiO_2_ were synthesized and tested for the NH_3_ SCR of NO. The EPR investigations revealed that the catalytic activity of POM supported on TiO_2_ is governed by a synergistic effect between the vanadium redox property and acidity of the supported catalyst. Alkali metal cations modulate accessibility and surface acidity, demonstrating that acidity strongly affects activity, whereas counter‐ion exchange has no effect on redox‐active vanadium. Thus, in the catalyst development for SCR reactions, it is crucial to optimize redox (electronic) properties and structural accessibility because larger cations hinder reducibility and block the active sites at the catalytic surface. These results were in accordance with the experimental data revealing that the catalytic activity of 7.5 wt.% XPV_3_/TiO_2_ (X = H, Na, K, Cs) in NH_3_‐SCR decreases with increasing basicity of the counter‐cation, consistent with prior findings on alkali and alkaline‐earth metals' deactivating effects. The observed reduction in apparent activation energy (E_a_) from H to K correlates with diminished ammonia adsorption due to surface acid site modification, with Cs displaying an anomalously high E_a_ likely due to limited redox properties. The rate constant at 250 °C (7.5 wt.% HPV_3_/TiO_2_) indicated a competitive catalytic performance relative to similar established catalysts (3 wt.% V_2_O_5_‐15 wt.% H_3_PMo_12_O_40_/TiO_2_) in the literature under similar conditions. Notably, all catalysts achieved 100 % N_2_ selectivity without N_2_O formation, suggesting their efficacy and environmental safety in NH_3_‐SCR applications.

## Supporting Information

The Supporting Information is accompanied by various additional results and analytical data of catalysts and reaction solutions. The authors have cited additional references within the Supporting Information.^[^ ^38^, ^39^, ^40^, ^41^, ^42^, ^43^, ^44^, ^45^ ^]^

## Conflict of Interest

The authors declare no conflict of interest.

## Supporting information



Supporting Information

## Data Availability

The data that support the findings of this study are available from the corresponding author upon reasonable request.
