# Supplementary material for: Influence of Countercations on the NH3‐SCR of NO Using Supported Vanadium‐Containing Polyoxometalate Catalysts
Source: Chemistry. 2025 Nov 2;31(68):e02561. doi: 10.1002/chem.202502561 (PMC12679341; doi:10.1002/chem.202502561)
Supplement: Supplementary file 1 — Supporting Information [file CHEM-31-e02561-s001.docx]

**Influence of countercations on the NH_3_-SCR of NO using supported vanadium-containing polyoxometalate catalysts.**

Samrin Shaikh^a^, Leonhard Schill^b^, Thomas Krøier Rønne-Nielsen^b^, Mariusz Grzegorz Kubus^b^. James N. McPherson^b^, Andreas Pawlig^a^, Huirong Li^b^, Maximillian J. Poller,^a^ Susanne Mossin^b^, Anders Riisager^b^, and Jakob Albert^a^

^a^ Institute of Technical and Macromolecular Chemistry, Universität Hamburg, Bundesstraße 45, 20146 Hamburg, Germany

^b^ Department of Chemistry, Technical University of Denmark, Kemitorvet, Building 207, 2800 Kgs. Lyngby, Denmark

* Contact details of the corresponding author: jakob.albert@uni-hamburg.de

This document contains 12 tables and 9 figures on 18 pages.

# **Synthesis of Na_6_PV_3_Mo_9_O_40_, K_5_HPV_3_Mo_9_O_40_ and Cs_3.5_H_2.5_PV_3_Mo_9_O_40_ catalyst.**

## **1.1 Na_6_PV_3_Mo_9_O_40_ x 9 H_2_O**

For the preparation of Na_6_PV_3_Mo_9_O_40_, 2.00 g (1.18 mmol,1 eq.) of H_3_PV_3_Mo_9_O_40_ were dissolved in 20 mL deionized water. 0.581 g (7.08 mmol, 6 eq.) of sodium acetate was dissolved in 5 mL of deionized water. Both solutions were combined and stirred for 10 min. Subsequently, the resulting acetic acid and solvent were removed under reduced pressure (180 bar, 80 °C).

**Yield: 2.04 g (94 %)**

**1.2 K_5_HPV_3_Mo_9_O_40_ x 7 H_2_O**

For the preparation of K_5_HPV_3_Mo_9_O_40_, 2.00 g (1.18 mmol,1 eq.) of H_3_PV_3_Mo_9_O_40_ were dissolved in 20 mL deionized water. 0.69 g (7.08 mmol, 6 eq.) of potassium acetate were dissolved in 5 mL deionized water. Both solutions were combined and stirred for 10 min. Subsequently the resulting acetic acid and solvent were removed under reduced pressure (180 mbar, 80 °C).

**Yield: 2.12 g (95 %)**

**1.3 Cs_3.5_H_2.5_PV_3_Mo_9_O_40_ x 8 H_2_O**

For the preparation of Cs_3.5_H_2.5_PV_3_Mo_9_O_40_, 2.00 g (1.18 mmol,1 eqv.) of H_3_PV_3_Mo_9_O_40_ were dissolved in 20 mL deionized water. To this solution, 1.19 g (7.08 mmol, 6 eqv.) of CsCl were added, resulting in the yellow precipitation of Cs_3.5_H_2.5_PV_3_Mo_9_O_40_. The product was filtered off, washed with deionized water (approx. 5 x 10 mL) and dried at 90 °C for 6 hours. **Yield 2.30 g (79 %)**

1. **Catalyst characterization**

**Inductively coupled plasma optical emission spectrometry (ICP-OES) analysis**

ICP-OES was used to determine the elemental composition of the synthesized vanadium-substituted HPA and counter cation substituted Keggin-polyoxometalates.

**Table S1**: Determination of the percentage for the elements Na, K, Mo, P and V via ICP-OES of H_6_PV_3_Mo_9_O_40_.

**ICP-OES.** Calculated for H_6_PV_3_Mo_9_O_40_: 51 % Mo, 9.02 % V, 1.83 % P.

Found for H_6_PV_3_Mo_9_O_40_: 49.9 % Mo, 10.90 % V, 1.79 % P.

Data normalized to Phosphorous: P/V/Mo ratio: 1.0/3.0/8.9

| Molar ratios | | |
| --- | --- | --- |
| Mo | P | V |
| 8.9 | 1 | 3 |

**Table S2**: Determination of the percentage for the elements Na, K, Mo, P and V via ICP-OES of Na_6_PV_3_Mo_9_O_40_.

**ICP-OES.** Calculated for Na_6_PV_3_Mo_9_O_40_: 7.56 % Na, 47.31 % Mo, 8.37 % V, 1.70 % P.

Found for Na_6_PV_3_Mo_9_O_40_: 6.80 % Na, 46.92 % Mo, 8.17 % V, 1.62 % P.

Data normalized to Phosphorous: Na/P/V/Mo ratio: 5.7/1.0/3.1/9.3

| Molar ratios | | | |
| --- | --- | --- | --- |
| Na | Mo | P | V |
| 5.7 | 9.3 | 1 | 3.1 |

**Table S3**: Determination of the percentage for the elements Na, K, Mo, P and V via ICP-OES of K_5_H PV_3_Mo_9_O_40_.

**ICP-OES.** Calculated for K_5_HPV_3_Mo_9_O_40_: 12.21 % K, 44.93 % Mo, 7.95 % V, 1.61 % P.

Found for K_5_HPV_3_Mo_9_O_40_: 11.30 % K, 38.10 % Mo, 9.49 % V, 1.85 % P.

Data normalized to Phosphorous: K/P/V/Mo ratio: 4.8/1.0/3.1/9.1

| Molar ratios | | | |
| --- | --- | --- | --- |
| K | Mo | P | V |
| 4.8 | 9.1 | 1 | 3.1 |

**Table S4**: Determination of the percentage for the elements Cs, V, Mo, P and Cl via ICP-OES of Cs_3.5_H_2.5_PV_3_Mo_9_O_40_.

**ICP-OES.** Calculated for Cs_3.5_H_2.5_PV_3_Mo_9_O_40_: 32.09 % Cs, 34.75 % Mo, 6.15 % V, 1.25 % P.

Found for Cs_3.5_H_2.5_PV_3_Mo_9_O_40_: 21.90 % Cs, 39.80 % Mo, 6.75 % V, 1.45 % P.

Data normalized to Phosphorous: Cs/P/V/Mo ratio: 3.5/1.0/2.8/8.9

| Molar ratios | | | | |
| --- | --- | --- | --- | --- |
| Cs | Mo | P | V | Cl |
| 3.5 | 8.9 | 1 | 2.8 | 220 ppm |

**ATR-FTIR Spectroscopy**

ATR-FTIR spectra were measured in attenuated total reflection (ATR) measurement mode using a QATR-S single-reflection ATR (with a diamond prism). From the obtained data, the baseline was corrected first, and the peaks were determined manually.


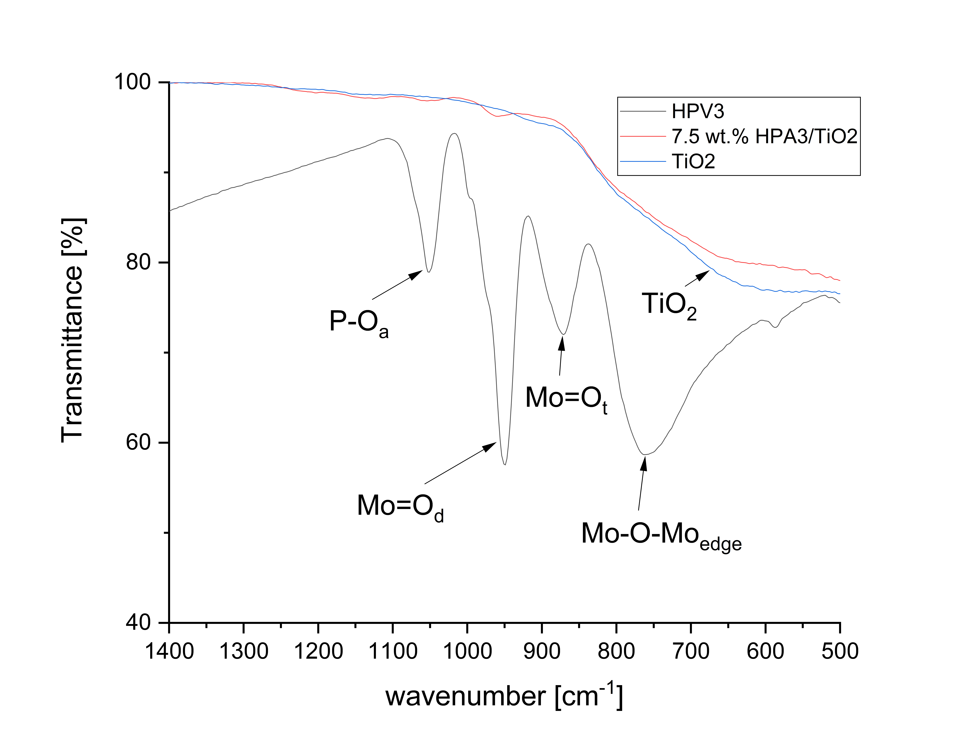


**V-O_b_-Mo**

V-O_b_-Mo

**Figure S1**: FTIR (ATR) spectrum of H_6_PV_3_Mo_9_O_40_ x 12 H_2_O. Vibration modes: 1048 Cm^-1^ (P-O), 947 Cm^-1^ (M=O_t_), 869 Cm^-1^ (M-O-M_vertex_), 740 cm^-1^ (V-O_b_-Mo), 728 Cm^-1^ (M-O-M_edge_), 3189 Cm^-1^ (Hydration water).


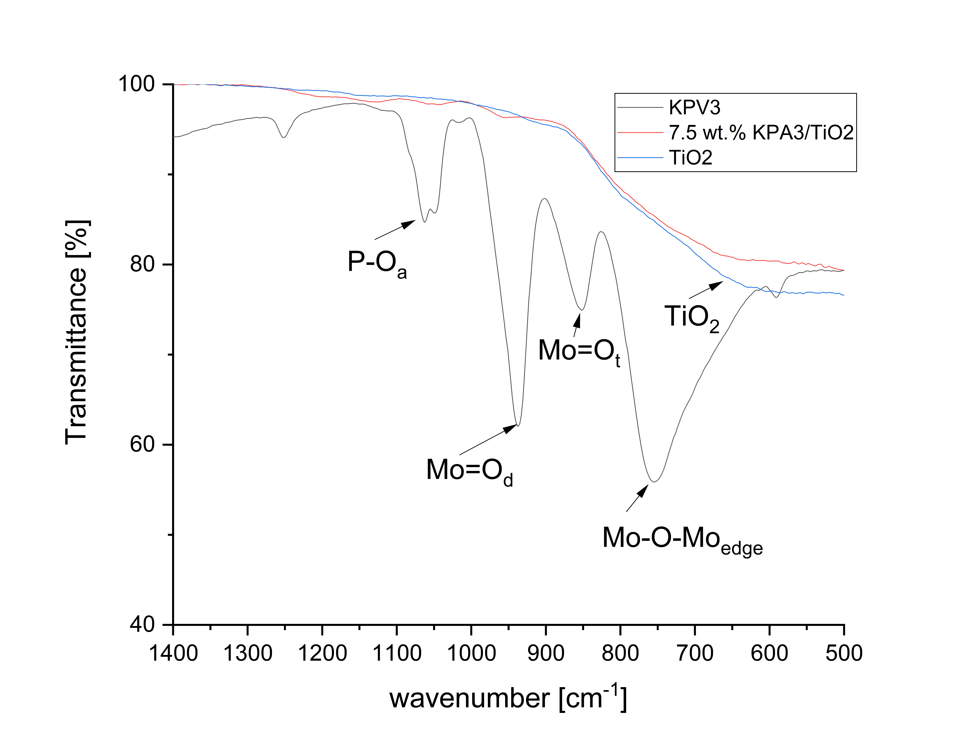
**Figure S2:** FTIR (ATR) spectrum of K_5_HPV_3_Mo_9_O_40_ x 9 H_2_O*.* Vibration modes: 1063 Cm^-1^ (P-O), 937 m^-1^ (M=O_t_), 851 Cm^-1^ (M-O-M_vertex_), 740 cm^-1^ (V-O_b_-Mo), 751 Cm^-1^ (M-O-M_edge_), 3420 Cm^-1^ (Hydration water).

**V-O_b_-Mo**

**V-O_b_-Mo**


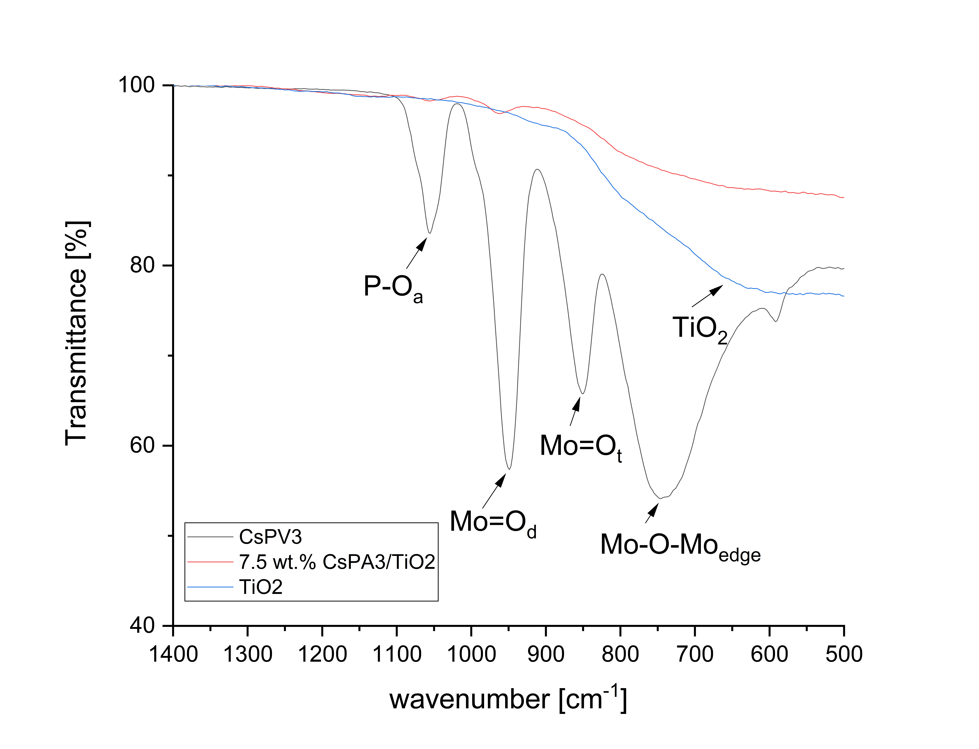


**V-O_b_-Mo**

## **Figure S3**: FTIR (ATR) spectrum of Cs_3.5_H_2.5_PV_3_Mo_9_O_40_ x 8 H_2_O. Vibration modes: 1048 Cm^-1^ (P-O), 947 *c*m^-1^ (M=O_t_), 869 *c*m^-1^ (M-O-M_vertex_), 728 *c*m^-1^ (M-O-M_edge_), 740 cm^-1^ (V-O_b_-Mo), 3189 *c*m^-1^ (Hydration water).


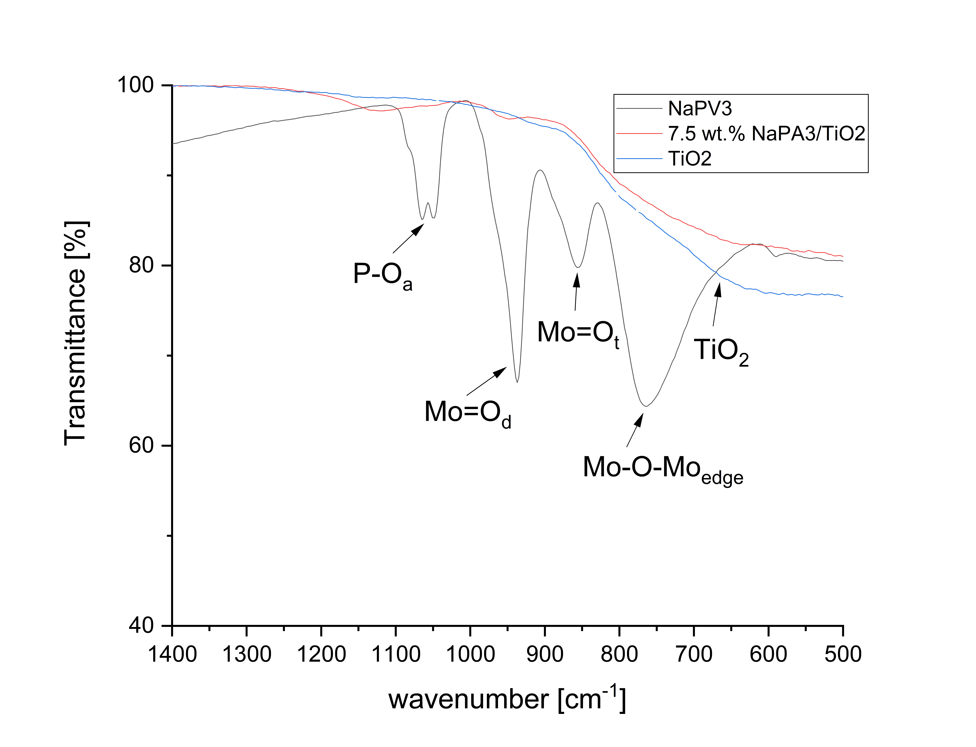


**V-O_b_-Mo**

**Figure S4**: FTIR (ATR) spectrum of Na_6_PV_3_Mo_9_O_40_ x 9 H_2_O. Vibration modes: 1048 cm^-1^ (P-o), 950 cm^-1^ (M=O_t_), 845 cm^-1^ (M-O-M_vertex_),740 cm^-1^ (V-O_b_-Mo), 758 cm^-1^ (M-O-M_edge_), 3307 cm^-1^ (Hydration water).


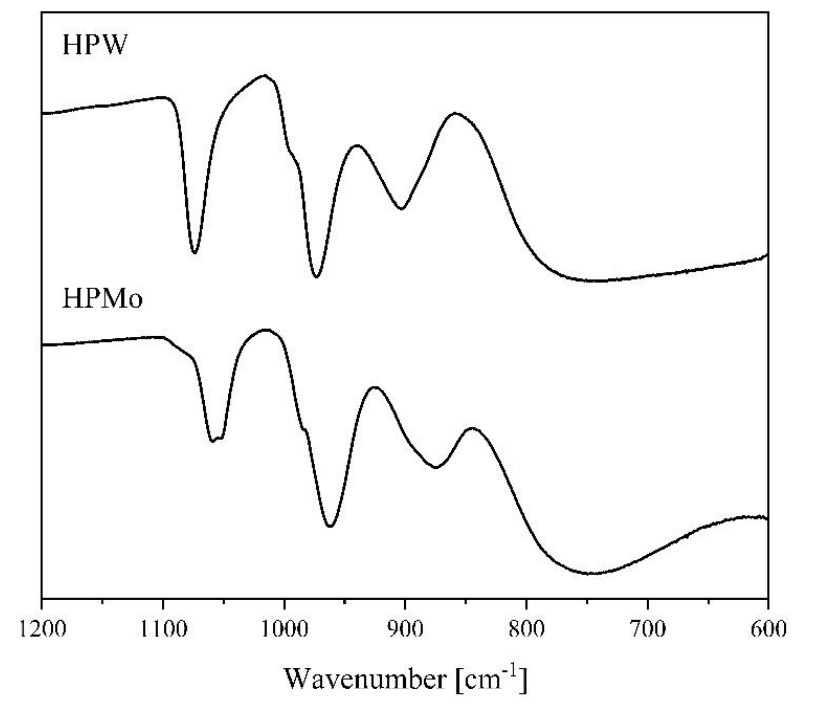


**Figure S4a**: Comparative FTIR (ATR) spectrum of HPMo and HPW.^(1)^

**X-ray diffraction patterns**


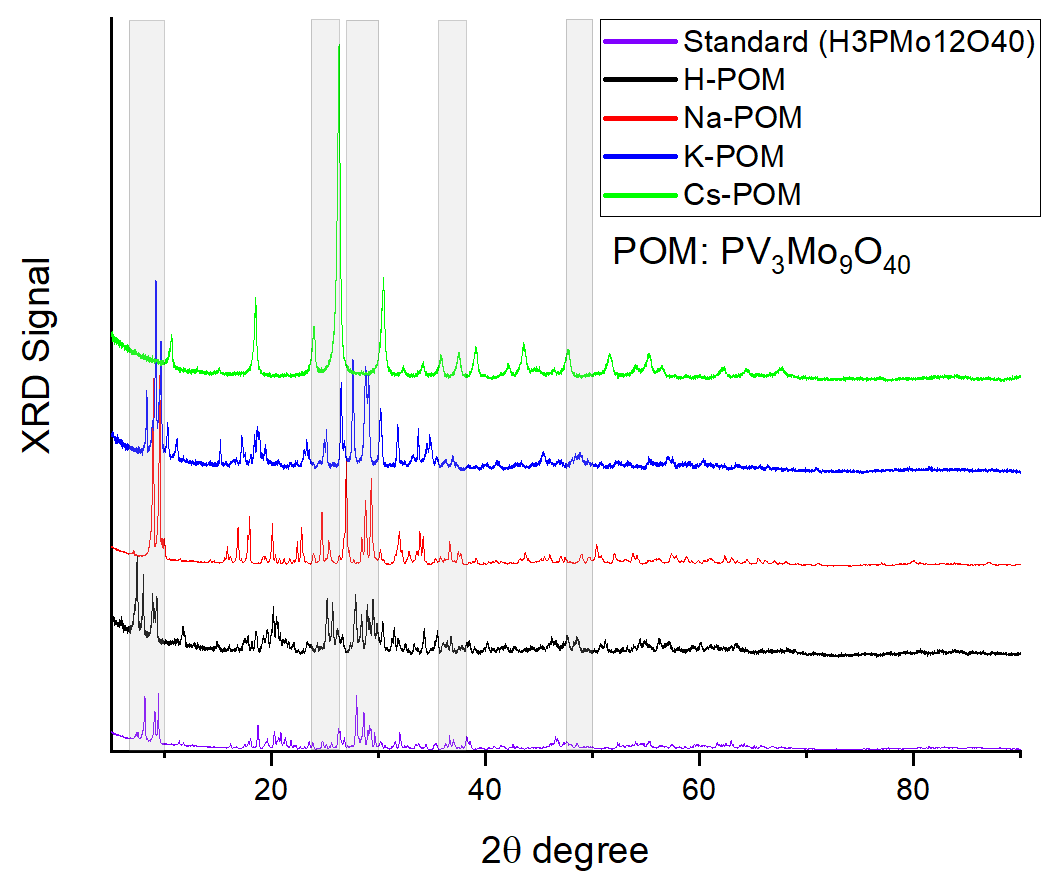


**Fig.S5** XRD spectra of X[POM]/TiO_2_, (X= H, Na, K, Cs).

**TGA analysis**


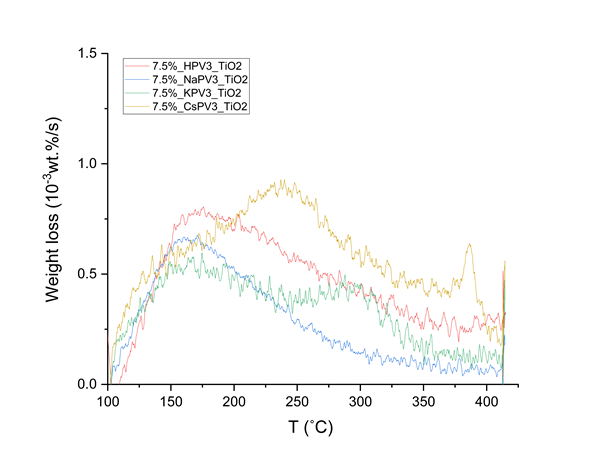


**Figure S6**: TGA analysis of the synthesized POMs supported on anatase.

**NH_3_-Temperature Programmed Desorption**

**Table S5: Relative number of acid sites as measured by NH_3_-TPD**

| **7.5 wt. % Catalyst** | **100 -200 °C** | **200 - 300 °C** | **>300 °C** | **Total** | **Total Loss** | **Cation/Mo**  **XPS**  **[mol/mol]** | **Cation/Mo**  **ICP**  **[mol/mol]** |
| --- | --- | --- | --- | --- | --- | --- | --- |
| **H-POM/TiO_2_** | **11.1** | **28.8** | **60.1** | **100.0** | **0.0** | -- | -- |
| Na-POM/TiO_2_ | 3.6 | 7.2 | 7.3 | 18.1 | 81.9 | 0.62 | 0.60 |
| K-POM/TiO_2_ | 4.7 | 7.9 | 0.7 | 13.3 | 86.7 | 0.65 | 0.73 |
| Cs-POM/TiO_2_ | 6.1 | 22.2 | 22.1 | 50.3 | 49.7 | 0.26 | 0.40 |
| TiO2 | 1.2 | 2.2 | 4.4 | 7.8 | - | - | - |

**Loss of acid sites is more pronounced for the strong acid sites:** (e.g. the Cs-POM/TiO_2_ retained almost all sites in the range 200 -300 °C while it lost almost 2/3 of the strong (>300 °C) sites as compared to the H-POM. Similarly, the K-POM retained almost a third of the sites in the range 200 – 300 °C, while it lost almost all of the strong sites.). The preferential binding of alkali metals to strong acid sites is in line with previous reports^2-3^.

**Cs-POM retains significantly more acid sites:** The number of acid sites on the Cs containing catalyst is 50.3 % of the corresponding number over the H-POM/TiO_2_. The Na and KPOM based catalysts both retain less than 20 % of the acid sites. This difference reflects the incomplete cation exchange observed for the Cs sample as determined by XPS and ICP. The relative loss of acid sites for the Cs containing sample is about 41 % lower than the average of the Na and K-containing samples, roughly matching the relative differences in cation/Mo ratios of 59 and 40 % according to XPS and ICP, respectively. This might hint at the loss of one or slightly above one acid site per cation in the studied cation concentration range.

**Continuous rotation electron diffraction microscopy (3D-ED)**

Continuous rotation 3D electron diffraction (3D-ED) data for H_6_[Mo_9_V_3_PO_40_]•3(H_2_O) and K_5_H[Mo_12_PO_40_]•7(H_2_O) were collected using a Rigaku XtaLAB Synergy-ED dedicated electron diffractometer, equipped with a Rigaku Oxford Diffraction HyPix detector.^(4-5)^ Microcrystalline samples were ground gently between two glass slides and loaded onto a continuous carbon film TEM grid, which was then inserted into the electron diffractometer.

Data acquisition for H_6_[Mo_9_V_3_PO_40_]•3(H_2_O) was performed at ambient temperature using an electron wavelength of 0.0251 Å (200 keV), with continuous rotation of the crystal around a single axis (−75° ≤ *a* ≤ 60°) at 3 s/° (total time: 6 min and 45 s, total dose: 1.5 × 10^1^ e/Å^2^) inside a selected area (with an effective diameter of ~1.9 mm).

Data acquisition for K_5_H[Mo_12_PO_40_]•7(H_2_O) was performed at 100 K using an electron wavelength of 0.0251 Å (200 keV), with continuous rotation of the crystal around a single axis (−60° ≤ *a* ≤ 60°) at 5 s/° (total time: 10 min, total dose: 5.5 e/Å^2^) inside a 100 µm selected area.

The raw diffraction images were processed using CrysAlisPro,^6^ and the structure was solved by ShelXT^7^ using intrinsic phasing. The structural model was refined by ShelXL ^8^using a kinematical approximation in the Olex2 ^9^graphical user interface using published scattering factors.

During refinenement of H_6_[Mo_9_V_3_PO_40_]•3(H_2_O), rigid body restraints (RIGU) were applied, and an extinction correction (EXTI) was used to reduce the impacts of dynamical scattering events. Two outlying reflections were omitted under the assumption that they were particularly affected by multiple scattering events. The metal site was modelled as Mo (75 % occupancy) and V (25 % occupancy), and the locations of the metals were freely refined. A strong SIMU restraint was applied to the two metal sites to keep the overlapping anisotropic displacement parameters stable during refinenement. A water solvent molecule was located in the difference map, and its hydrogen atoms were placed on riding positions that were disordered over four locations (related by symmetry). Crystal data and structure refinement parameters are summarised below in **Table S7.** The six protons per formula unit which are presumed to balance the charge of the [Mo_9_V_3_PO_4_]^6−^ anion could not be located in the structure.

A summary of the crystal data and structure refinements for K_5_H [Mo_12_PO_40_]•7(H_2_O) (CCDC number: 2453881) is given in **Tables S8-S11**. An extinction correction was applied during the kinematic refinements of electron diffraction data. Due to difficulties in the determination of the accurate orientation of hydrogen bonds in the structure, hydrogen atoms of water molecules were not included in the structural model. The occupancies of K atoms were refined freely and after stabilization, fixed in the final stages. ISOR restraint was applied for O6 and O7 atoms, and RIGU restraint for P1-O-O7 central cluster unit in the structure refinement.

| **Table S6.** Metric parameters from the 3D-ED structure of [Mo_9_V_3_PO_40_]^6−^•3(H_2_O). | | |
| --- | --- | --- |
|  | M = Mo | M = V |
| {PO_4_} |  |  |
| P1—O1 / Å: | 1.618(14) | |
| O1—P1—O1* / °: | 109.5(7) | |
|  |  |  |
| {MO_6_} |  |  |
| M—O1 / Å: | 2.596(13) | 2.78(6) |
| M—O2 / Å: | 1.94(3) | 1.88(6) |
| M—O3 / Å: | 2.132(15) | 2.34(4) |
| M—O4 / Å: | 1.75(2) | 1.48(7) |
| 12 *cis* O—M—O angles: |  |  |
| O1—M—O2 / °: | 58.8(8) | 55.0(19) |
| O1—M—O3 / °: | 101.7(9) | 91.3(19) |
| O2—M—O2* / °: | 84.5(12) | 87(3) |
| O2—M—O3 / °: | 88.9(11) | 85.1(14) |
| O3—M—O3* / °: | 89(2) | 79(3) |
| O2—M—O4 / °: | 103.8(11) | 119(2) |
| O3—M—O4 / °: | 96.6(12) | 97(3) |
| 3 *trans* O—M—O angles: |  |  |
| O1—M—O4 / °: | 154.4(13) | 170(3) |
| O2—M—O3 / °: | 159.6(11) | 142(3) |
|  |  |  |
| Other: |  | |
| O4…O5W / Å: | 3.27(2) | |
| P1—O1—M / °: | 123.8(12) | 130.6(15) |
| * denotes the same atom at a symmetry-equivalent position. | | |

| **Table S7.** Crystal data and structure refinement for [Mo_9_V_3_PO_40_]^6−^•3(H_2_O). | |
| --- | --- |
| Identifikation code (**CCDC**) | exp_702 (**2492701**) |
| Empirical formula | [Mo_9_V_3_PO_40_]^6−^•3(H_2_O) |
| Formula weight | 1741.27 |
| Temperature/K | 293(2) |
| Crystal system | cubic |
| Space group | I-43m |
| a/Å | 12.3896(10) |
| b/Å | 12.3896(10) |
| c/Å | 12.3896(10) |
| α/° | 90 |
| β/° | 90 |
| γ/° | 90 |
| Volume/Å^3^ | 1901.8(5) |
| Z | 2 |
| *ρ*_calc_g/cm^3^ | 3.041 |
| *μ*/mm^−1^ | 0.000 |
| F(000) | 422.0 |
| Crystal size/mm^3^ | 0.0006 × 0.0003 × ? |
| Radiation | electron (*λ* = 0.0251) |
| 2*θ* range for data collection/° | 0.164 to 1.79 |
| Index ranges | −14 ≤ *h* ≤ 14, −15 ≤ *k* ≤ 15, −15 ≤ *l* ≤ 15 |
| Reflections collected | 5291 |
| Independent reflections | 393 [R_int_ = 0.1942, R_sigma_ = 0.0729] |
| Data/restraints/parameters | 393/54/39 |
| Goodness-of-fit on F^2^ | 1.522 |
| Final R indexes [I ≥ 2σ (I)] | R_1_ = 0.1346, wR_2_ = 0.3704 |
| Final R indexes [all data] | R_1_ = 0.1469, wR_2_ = 0.3873 |
| Largest diff. peak/hole / e Å^−3^ | 0.21/−0.25 |

| Table S8. Crystal data and structure refinement for K_6_[Mo_12_PO_40_]•7(H_2_O). | |
| --- | --- |
| Identification code (CCDC) | exp_1118_autored (**2453880**) |
| Empirical formula | K_5.44_Mo_12_O_47_P |
| Formula weight | 2146.90 |
| Temperature/K | 100.0(1) |
| Crystal system | orthorhombic |
| Space group | Pnnm |
| a/Å | 17.181(4) |
| b/Å | 11.6217(16) |
| c/Å | 10.738(2) |
| α/° | 90 |
| β/° | 90 |
| γ/° | 90 |
| Volume/Å^3^ | 2144.2(8) |
| Z | 2 |
| ρ_calc_g/cm^3^ | 3.325 |
| μ/mm^‑1^ | 0.000 |
| F(000) | 540.0 |
| Crystal size/mm^3^ | 0.00012 × 0.0001 × 0.0001 |
| Radiation | electron (λ = 0.0251) |
| 2Θ range for data collection/° | 0.15 to 1.796 |
| Index ranges | -21 ≤ h ≤ 21, -14 ≤ k ≤ 14, -13 ≤ l ≤ 13 |
| Reflections collected | 10609 |
| Independent reflections | 2320 [R_int_ = 0.1965, R_sigma_ = 0.1863] |
| Data/restraints/parameters | 2320/21/180 |
| Goodness-of-fit on F^2^ | 1.855 |
| Final R indexes [I>=2σ (I)] | R_1_ = 0.2211, wR_2_ = 0.4361 |
| Final R indexes [all data] | R_1_ = 0.2474, wR_2_ = 0.4480 |
| Largest diff. peak/hole / e Å^-3^ | 0.71/-0.51 |

**Table S9. Selected Atomic Occupancy for K_5_H [Mo_12_PO_40_]•7(H_2_O).**

| Atom | *Occupancy* |  |
| --- | --- | --- |
| K1 | 0.57 |  |
| K3 | 0.74 |  |
| K4 | 0.78 |  |

| Table S10. Selected Bond Lengths for K_5_H [Mo_12_PO_40_]•7(H_2_O). | | | | | | |
| --- | --- | --- | --- | --- | --- | --- |
| Atom | **Atom** | **Length/Å** |  | **Atom** | **Atom** | **Length/Å** |
| Mo1 | O3 | 1.866(12) |  | Mo3 | O10 | 1.97(2) |
| Mo1 | O2 | 1.67(2) |  | Mo3 | O6 | 2.445(19) |
| Mo1 | O12 | 1.895(13) |  | Mo4 | O4 | 1.658(17) |
| Mo1 | O7 | 2.40(2) |  | Mo4 | O11 | 1.978(11) |
| Mo2 | O13 | 1.641(15) |  | Mo4 | O3 | 1.901(16) |
| Mo2 | O11 | 1.851(12) |  | Mo4 | O5 | 1.896(19) |
| Mo2 | O8 | 1.930(17) |  | Mo4 | O10 | 1.828(15) |
| Mo2 | O12 | 1.814(17) |  | Mo4 | O6 | 2.39(2) |
| Mo2 | O5 | 1.91(2) |  | Mo4 | O7^2^ | 2.492(19) |
| Mo2 | O6 | 2.44(2) |  | P1 | O6 | 1.574(19) |
| Mo2 | O7 | 2.472(19) |  | P1 | O7 | 1.53(2) |
| Mo3 | O9 | 1.651(14) |  | K4 | O14 | 2.38(4) |
| Mo3 | O8 | 1.834(19) |  |  |  |  |

| Table S11. Selected Bond Angles for K_5_H [Mo_12_PO_40_]•7(H_2_O). | | | | | | | | |
| --- | --- | --- | --- | --- | --- | --- | --- | --- |
| Atom | Atom | Atom | Angle/˚ |  | Atom | Atom | Atom | Angle/˚ |
| O3 | Mo1 | O3^1^ | 92.6(10) |  | O9 | Mo3 | O8^1^ | 102.9(9) |
| O3 | Mo1 | O12^1^ | 86.0(6) |  | O9 | Mo3 | O10 | 97.4(8) |
| O3^1^ | Mo1 | O12^1^ | 155.4(10) |  | O9 | Mo3 | O6 | 157.3(5) |
| O3 | Mo1 | O7^2^ | 94.7(9) |  | O8 | Mo3 | O8^1^ | 88.4(14) |
| O3 | Mo1 | O7^3^ | 64.8(8) |  | O8^1^ | Mo3 | O10 | 87.4(8) |
| O2 | Mo1 | O3 | 99.6(9) |  | O8 | Mo3 | O10 | 159.7(10) |
| O2 | Mo1 | O12 | 104.8(9) |  | O8^1^ | Mo3 | O6 | 95.8(10) |
| O2 | Mo1 | O7^3^ | 159.4(5) |  | O8 | Mo3 | O6 | 64.5(8) |
| O12 | Mo1 | O12^1^ | 85.2(8) |  | O10^1^ | Mo3 | O10 | 89.7(15) |
| O12^1^ | Mo1 | O7^3^ | 62.6(8) |  | O10 | Mo3 | O6 | 96.2(9) |
| O12^1^ | Mo1 | O7^2^ | 90.8(8) |  | O10 | Mo3 | O6^1^ | 64.5(8) |
| O7^3^ | Mo1 | O7^2^ | 41.0(9) |  | O6 | Mo3 | O6^1^ | 44.9(9) |
| O13 | Mo2 | O11 | 100.3(9) |  | O4 | Mo4 | O11^1^ | 101.6(8) |
| O13 | Mo2 | O8^2^ | 103.5(10) |  | O4 | Mo4 | O3 | 99.6(9) |
| O13 | Mo2 | O12 | 104.1(9) |  | O4 | Mo4 | O5 | 103.3(11) |
| O13 | Mo2 | O5^2^ | 101.4(11) |  | O4 | Mo4 | O10^1^ | 99.5(10) |
| O13 | Mo2 | O6^2^ | 158.6(9) |  | O4 | Mo4 | O6 | 160.8(7) |
| O13 | Mo2 | O7^2^ | 158.2(10) |  | O4 | Mo4 | O7^3^ | 156.2(8) |
| O11 | Mo2 | O8^2^ | 155.8(9) |  | O11^1^ | Mo4 | O6 | 91.7(8) |
| O11 | Mo2 | O5^2^ | 84.4(8) |  | O11^1^ | Mo4 | O7^3^ | 63.7(6) |
| O11 | Mo2 | O6^2^ | 92.6(8) |  | O3 | Mo4 | O11^1^ | 85.5(6) |
| O11 | Mo2 | O7^2^ | 65.5(6) |  | O3 | Mo4 | O6 | 95.1(8) |
| O8^2^ | Mo2 | O6^2^ | 63.5(8) |  | O3 | Mo4 | O7^3^ | 62.3(8) |
| O8^2^ | Mo2 | O7^2^ | 92.6(9) |  | O5 | Mo4 | O11^1^ | 84.6(8) |
| O12 | Mo2 | O11 | 91.3(6) |  | O5 | Mo4 | O3 | 156.5(11) |
| O12 | Mo2 | O8^2^ | 86.5(8) |  | O5 | Mo4 | O6 | 64.0(10) |
| O12 | Mo2 | O5^2^ | 154.5(11) |  | O5 | Mo4 | O7^3^ | 94.2(11) |
| O12 | Mo2 | O6^2^ | 92.5(8) |  | O10^1^ | Mo4 | O11^1^ | 158.9(10) |
| O12 | Mo2 | O7^2^ | 61.7(8) |  | O10^1^ | Mo4 | O3 | 92.4(9) |
| O5^2^ | Mo2 | O8^2^ | 87.3(7) |  | O10^1^ | Mo4 | O5 | 89.3(8) |
| O5^2^ | Mo2 | O6^2^ | 62.7(10) |  | O10^1^ | Mo4 | O6 | 67.5(9) |
| O5^2^ | Mo2 | O7^2^ | 93.9(11) |  | O10^1^ | Mo4 | O7^3^ | 96.8(10) |
| O6^2^ | Mo2 | O7^2^ | 43.2(7) |  | O6 | Mo4 | O7^3^ | 43.0(7) |

^1^+X,+Y,1-Z; ^2^1-X,1-Y,1-Z; ^3^1-X,1-Y,+Z

**2. Catalytic NH_3_-SCR experiments**

| 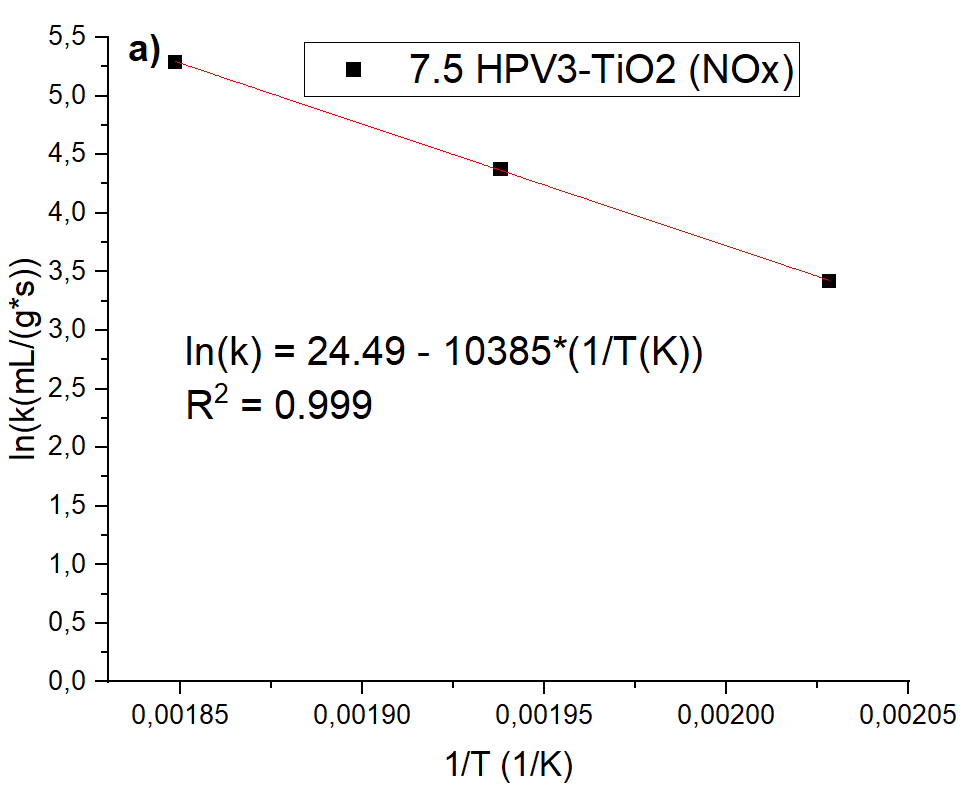 | 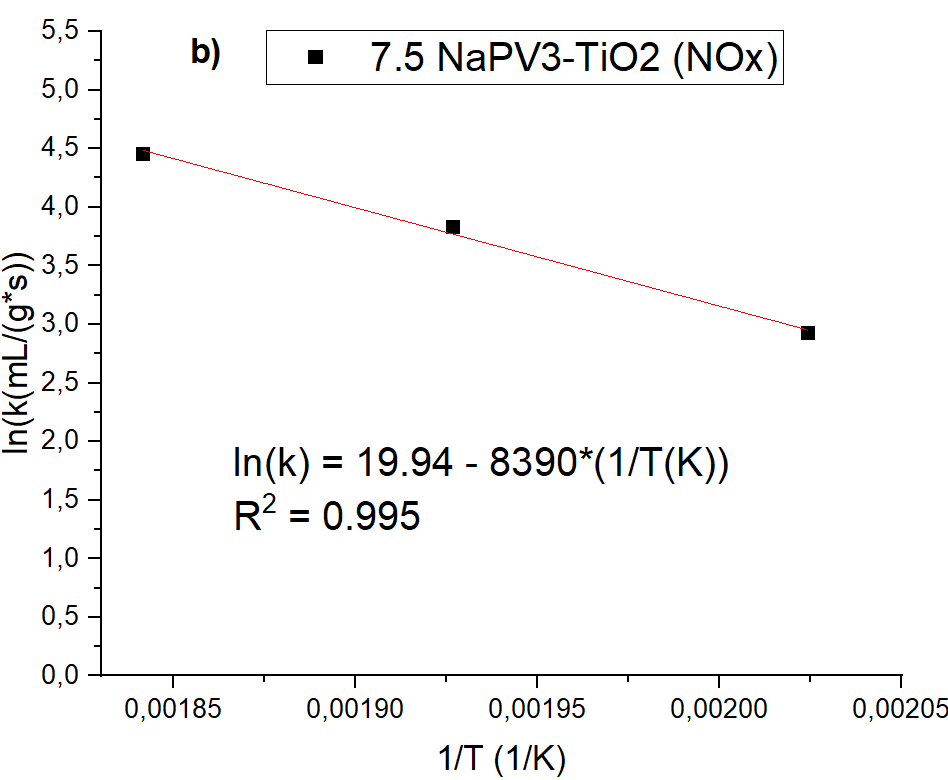 |
| --- | --- |
| 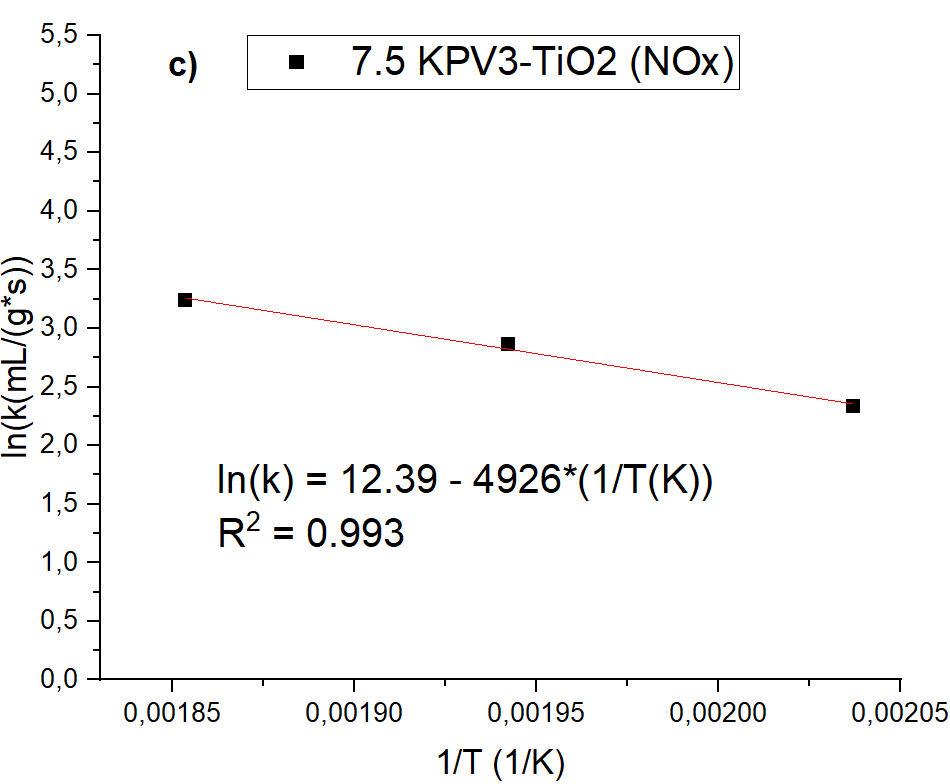 | 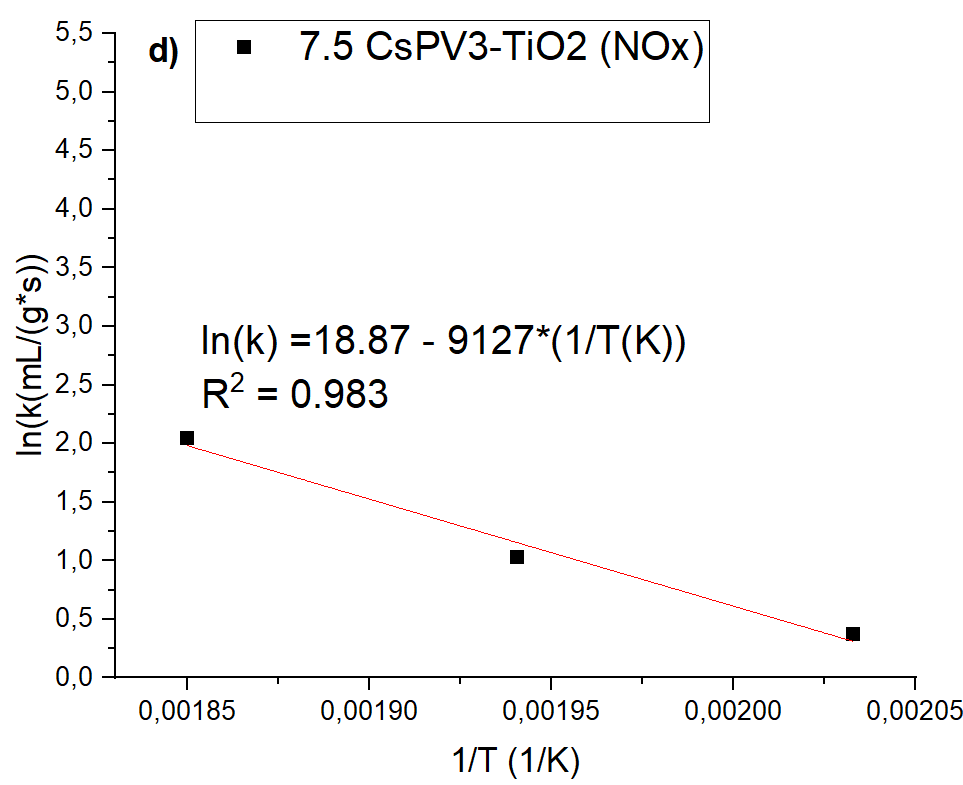 |

**Fig. S7:** Arrhenius plots derived from NO_x_ conversions at 215-275 ˚C over 7.5 wt.% PV3/TiO_2_ with different countercations.


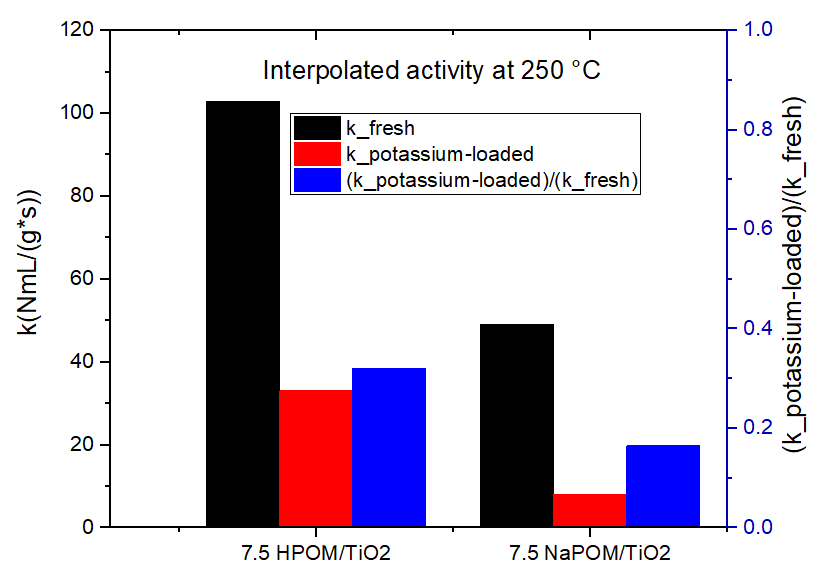


**Fig. S8** Effect of potassium (100 µmolK/g_cat_) on the activity of 7.5 HPOM/TiO_2_ and 7.5 NaPOM/TiO_2_ at 250 ˚C.

**Fig. S9** NO_x_ and NH_3_ conversions during NH_3_-SCR of K-poisoned (100 or 35 µmolK/g_cat_) 7.5 wt.% PV3/TiO_2_ with H and Na as countercations. Reaction conditions: [NO] = [NH_3_] = 600 ppm, [O_2_] = 4.5 vol%, balanced by N_2_, WHSV = 240,000 mL/(g·h).

**3. EPR experiments**

Low temperature EPR spectra were simulated using 3 species: Two well-defined species A and B with visible hyperfine splitting parameters. They are modeled using an axial spin Hamiltonian, see below. The third species C was modeled using an isotropic broad EPR spectrum.

The broadening is due to a larger variability in coordination environment at dynamic high temperature measurements and interactions with paramagnetic species. The gas flow contains both paramagnetic NO and paramagnetic O_2_ molecules that can contribute to the broadening.

Axial spin Hamiltonian:

$$H=g_{\parallel}\mu_{B}B_{z}+g_{\perp}\mu_{B}(B_{x}+B_{y})+A_{\parallel}S_{z}\cdot I_{z}+A_{\perp}(S_{x}\cdot I_{x}+S_{y}\cdot I_{y})$$

**Table S12: Spin Hamiltonian parameters used for simulating vanadium EPR spectra obtained at ex-situ conditions at 77K.**

| Catalyst | Type | $g_{\perp}$ | $g_{\parallel}$ | $g_{iso}$ | $A_{\perp}$  MHz | $A_{\parallel}$  MHz | Line width  (FWHM)  G | Distribution  % |
| --- | --- | --- | --- | --- | --- | --- | --- | --- |
| HPV3/TiO_2_ | A | 1.96 | 1.94 |  | 175 | 533 | 2.5 | 12.9 |
|  | B | 1.98 | 1.93 |  | 198 | 500 | 2 | 12.3 |
|  | C | - | - | 1.96 | - | - | 38 | 74.8 |
| NaPV3/TiO_2_ | A | 1.96 | 1.93 |  | 180 | 533 | 2 | 15.7 |
|  | B | 1.98 | 1.92 |  | 198 | 500 | 2 | 46.7 |
|  | C | - | - | 1.96 | - | - | 38 | 37.6 |
| KPV3/TiO_2_ | A | 1.96 | 1.93 |  | 180 | 533 | 2 | 14.4 |
|  | B | 1.98 | 1.92 |  | 198 | 500 | 2 | 41.3 |
|  | C | - | - | 1.96 | - | - | 38 | 44.3 |
| CsPV3/TiO_2_ | A | 1.96 | 1.93 |  | 180 | 533 | 2 | 11.0 |
|  | B | 1.98 | 1.92 |  | 198 | 500 | 2 | 5.9 |
|  | C | - | - | 1.96 | - | - | 38 | 83.1 |

For all samples species C dominated. It was also observed that for the HPV3 sample, species A and B were present in similar amounts, for NaPV3 and KPV3, species B was present in higher amount than A and for CsPV3 species A was present in higher amount than B. We cannot assign the species based on the current data alone, but it is clear that the counter ions have some influence on the prevailing coordination environment of V and that EPR at low temperature can distinguish between them.

For simulations of in-situ spectra, the parameters were fixed to the same values as in Table S8 with only minor adjustments, but the linewidths were increased, and the relative ratios of species were allowed to change as well. In general, the difference in features of species A and B were not resolved, and the observable hyperfine structure was merged between the two species. In addition, the amount of species C increased. At low temperature there was a cation-dependent distribution of species in the fresh samples, but this cannot be confirmed when measuring in-situ at reaction conditions due to the inherent lower resolution.

##### **References:**

1. J.C. Raabe, J. Aceituno Cruz, J. Albert, M.J. Poller, 2023. *Inorganics* 2023, **11**(4), p.138.
2. S. S. R. Putluru, S. B. Kristensen, J. Due-Hansen, A. Riisager, R. Fehrmann, *Catal. Today*, 2012, **184**, 192–196.
3. S. S. R. Putluru, L. Schill, A. Godiksen, R. Poreddy, S. Mossin, A. D. Jensen, R. Fehrmann, *Appl. Catal. B Environ.* 2016, **183**, 282–290.
4. K.N. Truong, S. Ito, J.M. Wojciechowski, C.R. Göb, C.J. Schürmann, A. Yamano, M. Del Campo, E. Okunishi, Y. Aoyama, T. Mihira, N. Hosogi, *Symmetry* 2023**, 15**, 1555 .
5. S. Ito, F. J. White, E. Okunishi, Y. Aoyama, A. Yamano, H. Sato, J. D. Ferrara, M. Jasnowski, M. Meyer, *CrystEngComm.* 2021, **23**, 8622–8630.
6. Rigaku Oxford Diffraction. CrysAlisPro Software System Version 1.171.44.86a (Rigaku Corporation, 2024 Wroclaw, Poland.
7. G. M. Sheldrick, *Acta Crystallogr. A.* 2015*,* **71**, 3–8 (**2015**).
8. O. V. Dolomanov, L. J. Bourhis, R. J. Gildea, J. A. K. Howard, H. Puschmann, *J. Appl. Crystallogr.* 2009*,* **42**, 339–341.
9. A. Saha, S. S. Nia, J. A. Rodríguez, *Chem. Rev*. 2022, **122**, 13883–13914.
